# Assessing the causal relationship between gut microbiota and diabetic nephropathy: insights from two-sample Mendelian randomization

**DOI:** 10.3389/fendo.2024.1329954

**Published:** 2024-03-18

**Authors:** Yipeng Fang, Yunfei Zhang, Qian Liu, Zenan Zheng, Chunhong Ren, Xin Zhang

**Affiliations:** ^1^ Laboratory of Molecular Cardiology, The First Affiliated Hospital of Shantou University Medical College, Shantou, Guangdong, China; ^2^ Laboratory of Medical Molecular Imaging, The First Affiliated Hospital of Shantou University Medical College, Shantou, Guangdong, China; ^3^ Shantou University Medical College, Shantou, Guangdong, China; ^4^ Tianjin Hospital, Tianjin, China; ^5^ Department of Cardiology, Binzhou Medical University Hospital, Binzhou, Shandong, China; ^6^ International Medical Service Center, The First Affiliated Hospital of Shantou University Medical College, Shantou, Guangdong, China; ^7^ Engineering Research Center of Key Technique for Biotherapy of Guangdong, Shantou, Guangdong, China

**Keywords:** causal inference, intestinal flora, diabetic kidney disease, Mendelian randomization, MiBioGen, eubacterium

## Abstract

**Background:**

The causal association between gut microbiota (GM) and the development of diabetic nephropathy (DN) remains uncertain. We sought to explore this potential association using two-sample Mendelian randomization (MR) analysis.

**Methods:**

Genome-wide association study (GWAS) data for GM were obtained from the MiBioGen consortium. GWAS data for DN and related phenotypes were collected from the FinngenR9 and CKDGen databases. The inverse variance weighted (IVW) model was used as the primary analysis model, supplemented by various sensitivity analyses. Heterogeneity was assessed using Cochran’s Q test, while horizontal pleiotropy was evaluated through MR-Egger regression and the MR-PRESSO global test. Reverse MR analysis was conducted to identify any reverse causal effects.

**Results:**

Our analysis identified twenty-five bacterial taxa that have a causal association with DN and its related phenotypes (p < 0.05). Among them, only the *g_Eubacterium_coprostanoligenes_group* showed a significant causal association with type 1 DN (p < Bonferroni-adjusted p-value). Our findings remained consistent regardless of the analytical approach used, with all methods indicating the same direction of effect. No evidence of heterogeneity or horizontal pleiotropy was observed. Reverse MR analysis did not reveal any causal associations.

**Conclusions:**

This study established a causal association between specific GM and DN. Our findings contribute to current understanding of the role of GM in the development of DN, offering potential insights for the prevention and treatment strategies for this condition.

## 1 Introduction

Diabetes mellitus (DM) presents a substantial socioeconomic and medical challenge due to its high prevalence and associated morbidity and mortality rates. In, 2019, the estimated global prevalence of DM was 9.3%, affecting 463 million individuals, with projections indicating a rise to 579 million by, 2030 and 700 million by, 2045 (1). Among the various complications of DM, diabetic nephropathy (DN) is one of the most prevalent, afflicting approximately 40% of DM patients (2). DN is now understood to be a debilitating and potentially fatal condition that significantly impacts patient quality of life and life expectancy. While hyperglycemia is recognized as the primary culprit, the underlying mechanisms behind DN development remain elusive. Furthermore, the pathophysiological changes associated with DN encompass hemodynamic abnormalities, inflammatory responses, and oxidative stress (3). Genetic factors, dietary choices, and lifestyle also play pivotal roles in the progression of DN. Recent research has shed light on the relationship between dysregulated gut microbiota (GM) and the onset and progression of DN (4, 5).

GM has emerged as a research hotspot in recent years. The concept of the gut-kidney axis, elucidating the intricate interplay between kidney disease and GM, was first introduced by Meijers BK et al. in, 2011 (6). There is a growing consensus suggesting a close relationship between intestinal dysbiosis and the occurrence of DM and its associated complications, including DN (7, 8). It was reported that over the last seven years, 124 articles focusing on GM and DN were published, and these continue to grow (9). The exploration of the underlying pathogenic mechanisms around obesity, inflammation, oxidative stress, metabolite imbalance, and the efficacy of Chinese herbal medicine is a hotspot for related research (9). The pathogenic role of GM and its metabolites in the development of DN is complex (3, 10). On the one hand, intestinal dysbiosis can induce persistent intestinal inflammation and hyperpermeability due to abnormal continuous stimulation (11). This, coupled with the depletion of beneficial metabolites and the accumulation of harmful ones resulting from intestinal dysbiosis, may contribute to kidney damage. Conversely, impaired renal function diminishes the excretion of harmful substances, potentially affecting the integrity of the intestinal barrier (12). Nitrogenous substances permeate the intestinal barrier due to increased permeability, consequently influencing the composition and function of GM. Moreover, GM plays an integral role in diabetes development by modulating various pathophysiological processes, including insulin secretion, insulin sensitivity, immune system regulation, and glycemic and lipid regulation (13, 14). Short chain fatty acids (SCFAs) deficiency is another important factor that may contribute to the progression of DN induced by intestinal dysbiosis (15). Cai KD et al. found a decrease of SCFAs concentration and SCFAs-related GM were found in patients with DN (16). Except for SCFAs, other metabolites of GM, like free fat acid, bile acid and Trimethylamine-N-oxide (TMAO), also play crucial role in the development of DN (15). These findings have stimulated research into the potential causal relationship between the intestinal dysbiosis and the development of DN.

To date, several observational studies have investigated the association between intestinal dysbiosis and DN (17–22). However, these studies are prone to inherent biases and reverse causation due to the inability to fully account for confounding factors. Consequently, establishing a causal link between GM and DN has remained a formidable challenge. The rapid advancement of Mendelian randomization (MR) analysis has provided a robust analytical tool for investigating causal relationships between GM and DN (23). As alleles are randomly inherited from parents to offspring during conception, a process akin to the random assignment of individuals to treatment and control groups in experiments, common confounding factors yield little effect over genetic variants. Consequently, by employing genetic variants as instrumental variables (IVs) to stand in for the exposure variable, MR analysis effectively nullifies the adverse effects of confounders commonly encountered in observational studies (24). This approach enhances the comprehensibility of findings and bolsters their credibility. In recent years, MR analysis has gained widespread recognition and application as a potent tool for exploring potential causal links between exposures and outcomes (23). Herein, we harnessed the capabilities of two-sample MR analysis to delve into the plausible causal connection between GM and the development of DN. Our research endeavors open up promising avenues for gaining fresh perspectives on early DN diagnosis and treatment centered around GM’s intricate role in the process.

## 2 Materials and methods

### 2.1 Ethical statement

Our current MR analysis used publicly available genome-wide association study (GWAS) datasets. Informed consent and ethical approval were obtained during the original GWAS study. As no new data were added, the need for ethical approval was waived for the present MR analysis.

### 2.2 Assumptions underlying MR

**Figure 1:**
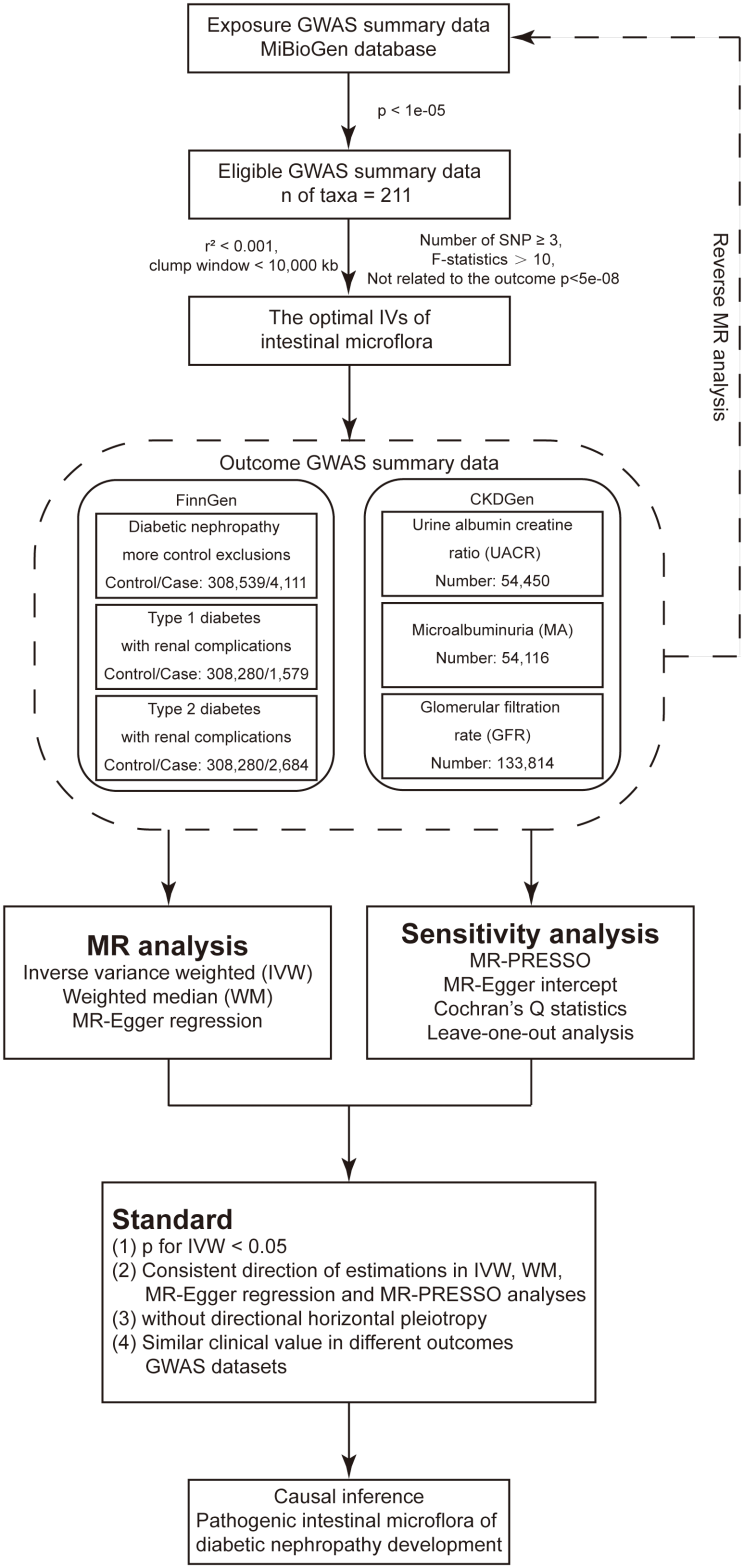
Flow chart of the present MR analysis.

The design of the present study was based on the three assumptions of MR as follows (25): (1) a strong and reliable association exists between the genetic variants and the risk factors (the gut bacteria); (2) there is no association between the genetic variants and confounding factors; (3) there are no alternative causal pathways linking the genetic variants to the outcome (the development of DN) other than through the risk factors under investigation. **Figure 1** shows the flow chart of the present MR analysis.

### 2.3 Exposure data of MR analysis

The GWAS summary statistics data used to identify potential proxy single nucleotide polymorphisms (SNPs) were sourced from the MiBioGen database, which originated from an extensive GWAS meta-analysis comprising 18,340 participants with 16S rRNA gene sequencing data (26). This dataset encompassed information collected from 24 population-based cohorts representing diverse ethnic groups, with participants of European descent (n=13,266), from the Middle East (n=481), Latin America (n=1,097), East Asia (n=811), the United States (n=114), and of multiple ancestral backgrounds (n=2,571). Following the exclusion of 15 taxa with unidentified classification (12 at the genus level and 3 at the family level), a total of 196 taxa were included in the final analysis, comprising 119 genera, 32 families, 20 orders, 16 classes, and 9 phyla.

### 2.4 Outcome data of MR analysis

Three GWAS summary statistics data for DN were obtained from the FinngenR9 database (https://r9.finngen.fi/) (27), including diabetic nephropathy more control exclusions (DM_NEPHROPATHY_EXMORE), type 1 diabetes with renal complications (T1DN, E4_DM1REN) and type 2 diabetes with renal complications (T2DN, E4_DM2REN). In addition, three DN-related phenotypes, including microalbuminuria (MA) (28), urine albumin creatine ratio (UACR) (28), and glomerular filtration rate (GFR) (29) in diabetic cohorts, were obtained from the CKDGEN database.

### 2.5 Selection of optimal IVs for exposure

Firstly, we included SNPs related to gut bacterial taxa in the MiBioGen database with a genome-wide significance threshold of p < 1×10^-05^, as reported in a previous study (30). Next, to minimize the effect of linkage disequilibrium (LD) on the results, the LD threshold and the window size for clumping were set to r^2^ < 0.001 and, 10000kb, respectively, based on the European-based 1,000 Genome Projects. To mitigate bias from weak IVs, SNPs with an F-statistics value less than 10 were excluded (F-statistics value = (β/SE)^2^) (31). Potential IVs closely related to the outcome phenotype were removed (p<5×10^-08^).

### 2.6 MR statistical analyses

Several outcome indicators, including the inverse variance weighted (IVW) method under random effects (32), weighted median (WM) (33), MR-Egger regression (34), and MR-PRESSO methods (35), were used to improve the robustness of our findings. Among these, the IVW method was used as the primary analysis, while the other three methods were used as adjuncts to IVW to verify the robustness of IVW results. During IVW analysis, which yields unbiased estimates, all SNPs considered in the study are deemed valid instrumental variables. During WM analysis, consistent effect estimates can be achieved even when over half of the weight assigned to the instruments contributing to the analysis is not considered valid. To evaluate potential directional pleiotropy, the MR-Egger regression and MR-Egger intercept analyses were employed (a p-value of less than 0.05 in the intercept analysis suggests horizontal pleiotropy). Heterogeneity was assessed using Cochran’s Q test with a significance threshold of p < 0.05. The criteria for detecting significant results were defined as follows (36): (1) the p-value for the IVW analysis was less than 0.05; (2) the direction of the effect obtained in the IVW, WM, MR-Egger regression, and MR-PRESSO methods should be consistent; (3) no significant horizontal pleiotropy was found in the MR-Egger intercept and MR-PRESSO analyses (p > 0.05); (4) the gut bacteria have similar clinical value in different significant phenotypes of DN. In the present study, we determined the statistical significance and potential association between gut bacteria and the risk of developing DN. A P value less than the Bonferroni adjusted IVW p (pFDR) cut-off was considered as statistically significant, while pFDR < p < 0.05 was interpreted as indicative of a potential link between gut bacteria and the susceptibility to developing DN. The pFDR value was 4.20×10^-04^(0.05/119) for the genus level, 1.56×10^-03^(0.05/32) for the family level, 2.50×10^-03^(0.05/20) for the order level, 3.13×10^-03^(0.05/16) for the class level and 5.56×10^-03^(0.05/9) for the phylum level. All results presented in our MR analysis were generated by the “TwoSampleMR” and “MRPRESSO” packages in R language software (version 4.3.1).

### 2.7 Reverse MR analysis

The reverse MR analysis was performed to detect the potential causal inference of DN development on the identified gut bacteria. In this part, DN and its phenotypes were considered as exposures, while the identified gut bacteria were considered as outcomes. SNPs extracted from the GWAS data related to DN and its phenotypes were identified as IVs. The criteria for identifying significant outcomes were similar to those used in the MR analysis.

## 3 Results

### 3.1 Selection of optimal SNPs

A total of 13,671 SNPs (p < 1×10^-05^) were obtained from the MiBioGen database (shown in **Supplementary Table S1**). After excluding ineligible SNPs, the most suitable SNPs were included in the final analysis. Comprehensive information regarding proxy SNPs can be found in **Supplementary Tables S2**-**S7**.

### 3.2 MR analyses

**Figure 2:**
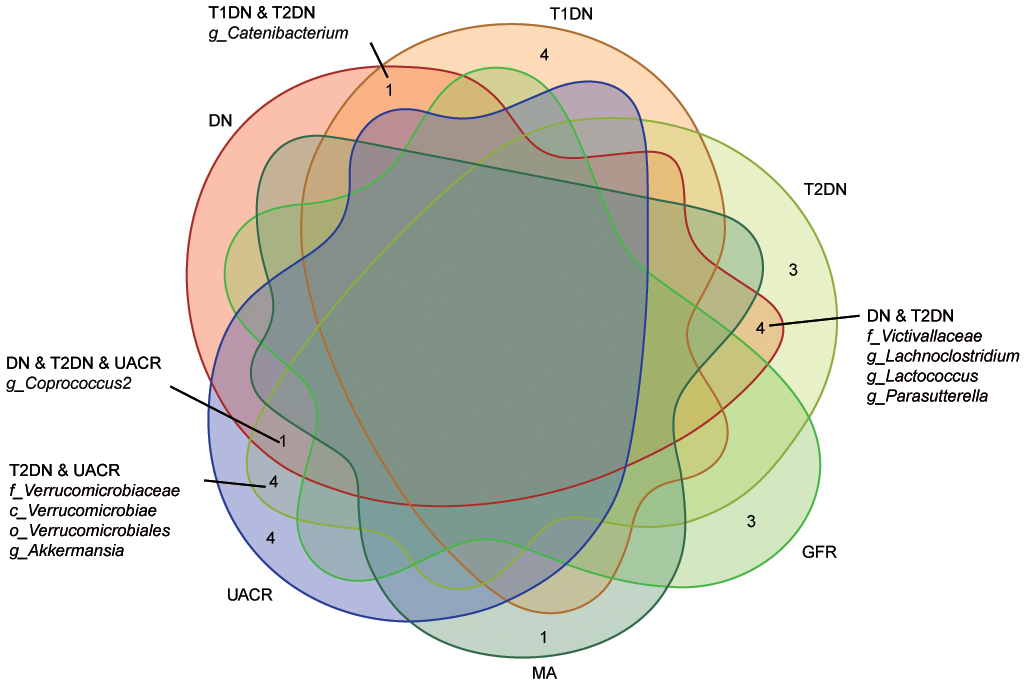
Venn diagram describes the significant findings obtained from different outcome GWAS data. Ten identified GMs were casually associated with the different kinds of phenotypes. DN, diabetic nephropathy; T1DN, type 1 diabetic nephropathy; T2DN, type 2 diabetic nephropathy; eGFR, estimated glomerular filtration rate; MA, microalbuminuria; UACR, urea albumin creatinine ratio.

**Figure 3:**
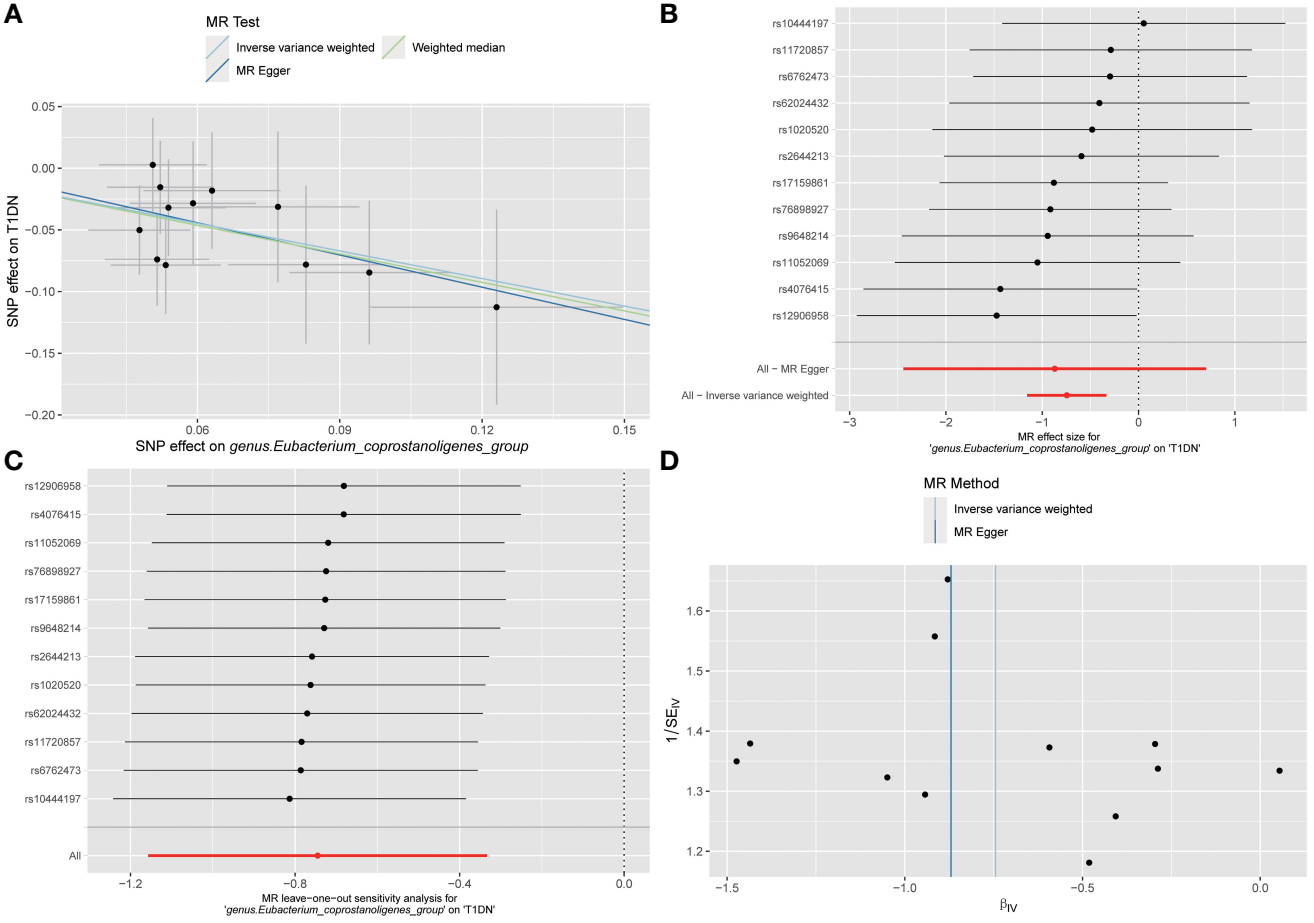
Visualization results for describing the causal association between *g_Eubacterium_coprostanoligenes_group* and the risk of developing T1DN. **(A)** scatter plot; **(B)** forest plot; **(C)** leave-one-out sensitivity analysis plot; **(D)** funnel plot. No significant outliers or publication bias found according to the visualization results.

The MR analyses (as outlined in **Table 1**) revealed that *g_Eubacterium_coprostanoligenes_group* exhibited a significantly negative association with the development of T1DN using the IVW method (OR = 0.475, 95% CI 0.314-0.717, p = 3.93×10^-04^). Moreover, another twenty-four GMs showed potential associations with the risk of developing DN in the IVW method (pFDR < p < 0.05). All these findings were consistent across WM, MR-Egger regression, and MR-PRESSO methods. **Table 2** summarizes the roles of the identified GMs in the development of DN across different phenotypes. The identified GMs mainly belong to the phyla *Bacteroidetes*, *Firmicutes*, *Proteobacteria*, and *Verrucomicrobia*. For a detailed breakdown of the MR analyses, please refer to **Supplementary Tables S8**-**S13**. Several GMs were demonstrated to be causally associated with different DN-related phenotypes, and we presented these results by venn diagrams (**Figure 2**). Scatter plots, leave-one-out sensitivity analysis plots, forest plots, and funnel plots illustrating the causal relationship between the identified GMs and DN development are presented in **Figure 3** (for *g_Eubacterium_coprostanoligenes_group*) and **Supplementary File S1** (for all identified GMs).

**Table 1:** Significant MR analysis results.

Outcome	Levels	Gut microbiota	SNP	IVW	p-value (IVW)	WM	MR-Egger	MR.PRESSO
DN	family	*Victivallaceae*	11	0.871(0.772-0.982)	0.025	0.937(0.791-1.109)	0.872(0.499-1.525)	0.871(0.787-0.964)
DN	genus	*Catenibacterium*	4	1.312(1.079-1.594)	0.006	1.352(1.067-1.712)	2.916(0.254-33.437)	1.312(1.204-1.429)
DN	genus	*Coprococcus2*	8	0.745(0.576-0.963)	0.025	0.705(0.506-0.981)	0.415(0.055-3.155)	0.745(0.604-0.919)
DN	genus	*Lachnoclostridium*	13	1.434(1.129-1.821)	0.003	1.414(1.005-1.989)	1.337(0.593-3.015)	1.434(1.167-1.763)
DN	genus	*Lactococcus*	8	0.851(0.73-0.992)	0.039	0.873(0.72-1.058)	0.615(0.304-1.243)	0.851(0.758-0.955)
DN	genus	*Parasutterella*	14	1.270(1.069-1.51)	0.007	1.288(1.026-1.618)	1.034(0.641-1.67)	1.270(1.132-1.426)
T1DN	genus	*Alistipes*	12	1.590(1.025-2.465)	0.038	1.421(0.778-2.594)	1.027(0.128-8.263)	1.590(1.057-2.391)
T1DN	genus	*Catenibacterium*	4	1.693(1.238-2.315)	0.001	1.67(1.115-2.501)	13.651(0.274-679.657)	1.693(1.287-2.228)
T1DN	genus	*Christensenellaceae_R_7_group*	8	1.700(1.026-2.816)	0.039	2.372(1.187-4.743)	4.110(0.697-24.239)	1.700(1.078-2.680)
T1DN	genus	*Eubacterium_coprostanoligenes_group*	12	0.475(0.314-0.717)	3.93e-04	0.463(0.260-0.823)	0.419(0.087-2.018)	0.475(0.365-0.617)
T1DN	genus	*Phascolarctobacterium*	8	0.527(0.352-0.79)	0.002	0.739(0.426-1.281)	0.115(0.019-0.697)	0.527(0.352-0.790)
T2DN	phylum	*Proteobacteria*	12	0.67(0.496-0.904)	0.009	0.685(0.460-1.022)	0.611(0.265-1.412)	0.670(0.510-0.879)
T2DN	class	*Betaproteobacteria*	10	0.624(0.449-0.869)	0.005	0.596(0.389-0.912)	0.478(0.168-1.364)	0.624(0.480-0.812)
T2DN	class	*Verrucomicrobiae*	11	1.51(1.097-2.078)	0.011	1.681(1.164-2.425)	1.054(0.347-3.199)	1.510(1.097-2.078)
T2DN	order	*Burkholderiales*	10	0.644(0.465-0.893)	0.008	0.591(0.380-0.919)	0.507(0.181-1.42)	0.644(0.478-0.868)
T2DN	order	*Verrucomicrobiales*	11	1.51(1.097-2.078)	0.011	1.681(1.164-2.426)	1.054(0.347-3.199)	1.510(1.097-2.078)
T2DN	family	*Verrucomicrobiaceae*	11	1.51(1.097-2.079)	0.011	1.681(1.156-2.444)	1.052(0.347-3.194)	1.510(1.097-2.079)
T2DN	family	*Victivallaceae*	11	0.836(0.711-0.983)	0.031	0.961(0.777-1.189)	0.785(0.355-1.736)	0.836(0.711-0.983)
T2DN	genus	*Akkermansia*	11	1.51(1.097-2.078)	0.011	1.683(1.164-2.434)	1.055(0.348-3.198)	1.51(1.097-2.078)
T2DN	genus	*Coprococcus2*	8	0.673(0.49-0.925)	0.015	0.641(0.420-0.976)	0.14(0.011-1.72)	0.673(0.496-0.913)
T2DN	genus	*Lachnoclostridium*	13	1.584(1.179-2.128)	0.002	1.347(0.909-1.997)	1.572(0.575-4.299)	1.584(1.266-1.981)
T2DN	genus	*Lactococcus*	8	0.802(0.648-0.993)	0.043	0.824(0.635-1.069)	0.41(0.166-1.015)	0.802(0.648-0.993)
T2DN	genus	*Parasutterella*	14	1.348(1.089-1.668)	0.006	1.41(1.055-1.884)	1.072(0.593-1.938)	1.348(1.205-1.507)
eGFR	family	*Peptostreptococcaceae*	4	0.951(0.904-1.000)	0.048	0.946(0.89-1.005)	0.948(0.868-1.035)	0.951(0.927-0.975)
eGFR	genus	*Anaerostipes*	4	1.081(1.009-1.158)	0.026	1.082(0.997-1.174)	1.180(0.845-1.649)	1.081(1.026-1.139)
eGFR	genus	*Eubacteriu_mxylanophilum_group*	5	1.109(1.037-1.186)	0.003	1.109(1.021-1.204)	1.056(0.847-1.317)	1.109(1.037-1.186)
MA	genus	*Anaerotruncus*	7	1.437(1.056-1.957)	0.021	1.431(0.944-2.171)	4.062(0.754-21.881)	1.437(1.103-1.873)
UACR	class	*Verrucomicrobiae*	4	1.585(1.014-2.48)	0.043	1.652(0.984-2.772)	1.762(0.005-671.997)	1.585(1.121-2.241)
UACR	order	*Verrucomicrobiales*	4	1.585(1.014-2.48)	0.043	1.652(0.976-2.796)	1.762(0.005-671.997)	1.585(1.121-2.241)
UACR	family	*Lactobacillaceae*	3	1.609(1.007-2.57)	0.047	1.412(0.767-2.601)	1.327(0.184-9.571)	N/A
UACR	family	*Verrucomicrobiaceae*	4	1.585(1.013-2.479)	0.044	1.652(0.932-2.927)	1.749(0.005-670.484)	1.585(1.121-2.241)
UACR	genus	*Akkermansia*	4	1.586(1.014-2.479)	0.043	1.652(0.957-2.851)	1.87(0.005-686.233)	1.586(1.122-2.24)
UACR	genus	*Clostridium_innocuum_group*	3	1.757(1.205-2.562)	0.003	1.552(0.870-2.767)	2.584(0.579-11.539)	N/A
UACR	genus	*Coprococcus2*	6	0.600(0.411-0.877)	0.008	0.644(0.387-1.071)	0.952(0.093-9.703)	0.581(0.37-0.911)
UACR	genus	*Holdemania*	7	0.707(0.513-0.972)	0.033	0.728(0.48-1.104)	0.596(0.122-2.916)	0.707(0.665-0.751)
UACR	genus	*Lactobacillus*	4	1.538(1.060-2.234)	0.024	1.355(0.845-2.173)	1.352(0.26-7.038)	1.538(1.069-2.214)

Abbreviation: MR, mendelian randomization; DN, diabetic nephropathy; T1DN, type 1 diabetic nephropathy; T2DN, type 2 diabetic nephropathy; eGFR, estimated glomerular filtration rate; MA, microalbuminuria; UACR, urea albumin creatinine ratio; SNP, single nucleotide polymorphism; IVW, inverse variance weighted; WM, weighted median.

N/A, Not applicable (due to insufficient number of SNPs).

**Table 2:** Summary of the role of the identified GM in DN developing.

Outcomes	Protective factor	Risk factor
DN	*f_Victivallaceae; g_Coprococcus2; g_Lactococcus*	*g_Catenibacterium; g_Lachnoclostridium; g_Parasutterella*
T1DN	*g_Eubacterium_coprostanoligenes_group; g_Phascolarctobacterium*	*g_Alistipes; g_Catenibacterium; g_Christensenellaceae_R_7_group*
T2DN	*p_Proteobacteria; c_Betaproteobacteria; o_Burkholderiales; f_Victivallaceae; g_Coprococcus2; g_Lactococcus*	*c_Verrucomicrobiae; o_Verrucomicrobiales; f_Verrucomicrobiaceae; g_Akkermansia; g_Lachnoclostridium; g_Parasutterella*
GFR	*g_Anaerostipes; g_Eubacterium_xylanophilum_group*	*f_Peptostreptococcaceae*
MA	N/A	*g_Anaerotruncus*
UACR	*g_Coprococcus2; g_Holdemania*	*c_Verrucomicrobiae; o_Verrucomicrobiales; f_Lactobacillaceae; f_Verrucomicrobiaceae; g_Akkermansia; g_Clostridium_innocuum_group; g_Lactobacillus*

Abbreviation: MR, mendelian randomization; DN, diabetic nephropathy; T1DN, type 1 diabetic nephropathy; T2DN, type 2 diabetic nephropathy; eGFR, estimated glomerular filtration rate; MA, microalbuminuria; UACR, urea albumin creatinine ratio.

N/A, Not applicable.

### 3.3 Directional horizontal pleiotropy and heterogeneity analyses

Analysis of MR-Egger regression intercepts indicated the absence of significant directional horizontal pleiotropy (as depicted in **Supplementary Table S14**, all p > 0.05). While some potential outliers among IVs for GMs were identified in leave-one-out plots (refer to **Supplementary File S1**), no significant outliers were detected according to the results of MR-PRESSO global test analyses (outlined in **Supplementary Table S15**, all p > 0.05). Furthermore, in both IVW and WM analyses, Cochran’s Q test did not reveal any significant heterogeneity among the IVs (detailed in **Supplementary Table S16**, all p > 0.05). In summary, our findings suggest the absence of significant directional horizontal pleiotropy and heterogeneity in the current MR analysis.

### 3.4 Reverse MR analysis

In the reverse MR analysis, it was observed that the development of T2DN exhibited a positive causal association with the abundance of *g_Lactococcus* in the IVW analysis (OR 1.094, 95% CI 1.009-1.185, p = 0.028). However, this causal effect was not consistently robust. The MR-Egger method yielded a negative association, although it was not statistically significant (OR 0.997, 95% CI 0.744-1.337, p = 0.989). Due to the limited number of valid SNPs associated with UACR (n = 2), a systematic analysis of the causal effect of UACR on the identified GMs could not be carried out. Overall, there is no substantial evidence that the development of DN causally affects the abundance of all identified GMs (see **Table 3**).

**Table 3:** Reverse MR analysis results.

Exposure	Levels	Gut microbiota	SNP	IVW	p-value (IVW)	WM	MR-Egger	MR.PRESSO
DN	family	*Victivallaceae*	16	1.021(0.964-1.081)	0.488	1.007(0.936-1.083)	0.972(0.88-1.074)	1.021(0.970-1.073)
DN	genus	*Catenibacterium*	13	1.033(0.966-1.105)	0.344	1.029(0.949-1.115)	1.005(0.897-1.126)	1.033(0.978-1.091)
DN	genus	*Coprococcus2*	17	0.995(0.963-1.029)	0.779	1.015(0.974-1.057)	0.993(0.936-1.053)	0.995(0.963-1.028)
DN	genus	*Lachnoclostridium*	17	1.016(0.987-1.046)	0.279	1.009(0.974-1.045)	1.002(0.951-1.055)	1.016(0.987-1.046)
DN	genus	*Lactococcus*	16	1.01(0.953-1.069)	0.743	1.01(0.939-1.087)	1.036(0.935-1.147)	1.010(0.953-1.069)
DN	genus	*Parasutterella*	17	1.008(0.974-1.043)	0.639	1.009(0.967-1.053)	1.022(0.961-1.086)	1.008(0.974-1.043)
T1DN	genus	*Alistipes*	11	0.998(0.972-1.025)	0.886	1.004(0.983-1.025)	1.005(0.959-1.053)	0.998(0.972-1.025)
T1DN	genus	*Catenibacterium*	10	1.022(0.983-1.062)	0.276	1.02(0.973-1.07)	1.011(0.946-1.08)	1.022(0.988-1.056)
T1DN	genus	*Christensenellaceae_R_7_group*	12	1.004(0.985-1.023)	0.689	1.012(0.99-1.035)	1.014(0.98-1.048)	1.004(0.985-1.023)
T1DN	genus	*Eubacterium_coprostanoligenes_group*	12	1.007(0.99-1.023)	0.419	1.008(0.987-1.03)	1.023(0.994-1.053)	1.007(0.995-1.018)
T1DN	genus	*Phascolarctobacterium*	12	1.007(0.982-1.033)	0.583	0.997(0.972-1.023)	0.992(0.948-1.038)	1.007(0.982-1.033)
T2DN	phylum	*Proteobacteria*	20	1.016(0.985-1.048)	0.306	1.01(0.966-1.055)	1.034(0.926-1.154)	1.016(0.993-1.040)
T2DN	class	*Betaproteobacteria*	20	0.996(0.965-1.028)	0.794	0.971(0.93-1.014)	0.978(0.873-1.096)	0.996(0.969-1.023)
T2DN	class	*Verrucomicrobiae*	20	1.023(0.98-1.068)	0.306	1.025(0.969-1.084)	1.051(0.898-1.231)	1.023(0.980-1.068)
T2DN	order	*Burkholderiales*	20	0.996(0.965-1.028)	0.799	0.973(0.932-1.017)	0.982(0.876-1.1)	0.996(0.969-1.024)
T2DN	order	*Verrucomicrobiales*	20	1.023(0.98-1.068)	0.306	1.025(0.971-1.082)	1.051(0.898-1.231)	1.023(0.980-1.068)
T2DN	family	*Verrucomicrobiaceae*	20	1.023(0.98-1.068)	0.306	1.025(0.969-1.084)	1.052(0.898-1.231)	1.023(0.980-1.068)
T2DN	family	*Victivallaceae*	20	0.999(0.925-1.079)	0.976	1.065(0.968-1.172)	1.281(0.991-1.655)	0.999(0.925-1.079)
T2DN	genus	*Akkermansia*	20	1.022(0.98-1.067)	0.310	1.024(0.969-1.083)	1.052(0.899-1.23)	1.022(0.980-1.067)
T2DN	genus	*Coprococcus2*	20	1.006(0.968-1.046)	0.748	1.014(0.96-1.07)	0.977(0.85-1.123)	1.006(0.974-1.039)
T2DN	genus	*Lachnoclostridium*	20	1(0.969-1.031)	0.997	1.005(0.963-1.049)	1.016(0.909-1.135)	1.000(0.977-1.023)
T2DN	genus	*Lactococcus*	20	1.094(1.009-1.185)	0.028	1.088(0.987-1.199)	0.997(0.744-1.337)	1.094(1.009-1.185)
T2DN	genus	*Parasutterella*	20	0.996(0.958-1.036)	0.854	1.014(0.963-1.069)	1.005(0.874-1.155)	0.996(0.960-1.034)
MA	genus	*AN/Aerotruncus*	6	0.958(0.882-1.041)	0.314	0.917(0.836-1.005)	0.901(0.708-1.146)	0.958(0.882-1.041)
UACR	class	*Verrucomicrobiae*	2	1.038(0.852-1.264)	0.710	N/A	N/A	N/A
UACR	order	*Verrucomicrobiales*	2	1.038(0.852-1.264)	0.710	N/A	N/A	N/A
UACR	family	*Lactobacillaceae*	2	0.954(0.805-1.131)	0.590	N/A	N/A	N/A
UACR	family	*Verrucomicrobiaceae*	2	1.038(0.853-1.263)	0.711	N/A	N/A	N/A
UACR	genus	*Akkermansia*	2	1.037(0.85-1.266)	0.717	N/A	N/A	N/A
UACR	genus	*Clostridium_innocuum_group*	2	1.001(0.803-1.249)	0.990	N/A	N/A	N/A
UACR	genus	*Coprococcus2*	2	1(0.875-1.143)	0.997	N/A	N/A	N/A
UACR	genus	*Holdemania*	2	0.897(0.773-1.041)	0.153	N/A	N/A	N/A
UACR	genus	*Lactobacillus*	2	0.94(0.793-1.116)	0.482	N/A	N/A	N/A
eGFR	family	*Peptostreptococcaceae*	6	1.059(0.983-1.14)	0.131	1.028(0.935-1.13)	0.94(0.769-1.147)	1.059(0.997-1.125)
eGFR	genus	*AN/Aerostipes*	6	0.957(0.891-1.029)	0.237	0.949(0.864-1.042)	0.863(0.711-1.048)	0.957(0.901-1.017)
eGFR	genus	*Eubacterium_xylanophilum_group*	6	0.973(0.886-1.069)	0.568	0.918(0.825-1.021)	0.976(0.735-1.295)	0.973(0.886-1.069)

Abbreviation: MR, mendelian randomization; DN, diabetic nephropathy; T1DN, type 1 diabetic nephropathy; T2DN, type 2 diabetic nephropathy; eGFR, estimated glomerular filtration rate; MA, microalbuminuria; UACR, urea albumin creatinine ratio; SNP, single nucleotide polymorphism; IVW, inverse variance weighted; WM, weighted median.

N/A, Not applicable (due to insufficient number of SNPs).

## 4 Discussion

**Figure 4:**
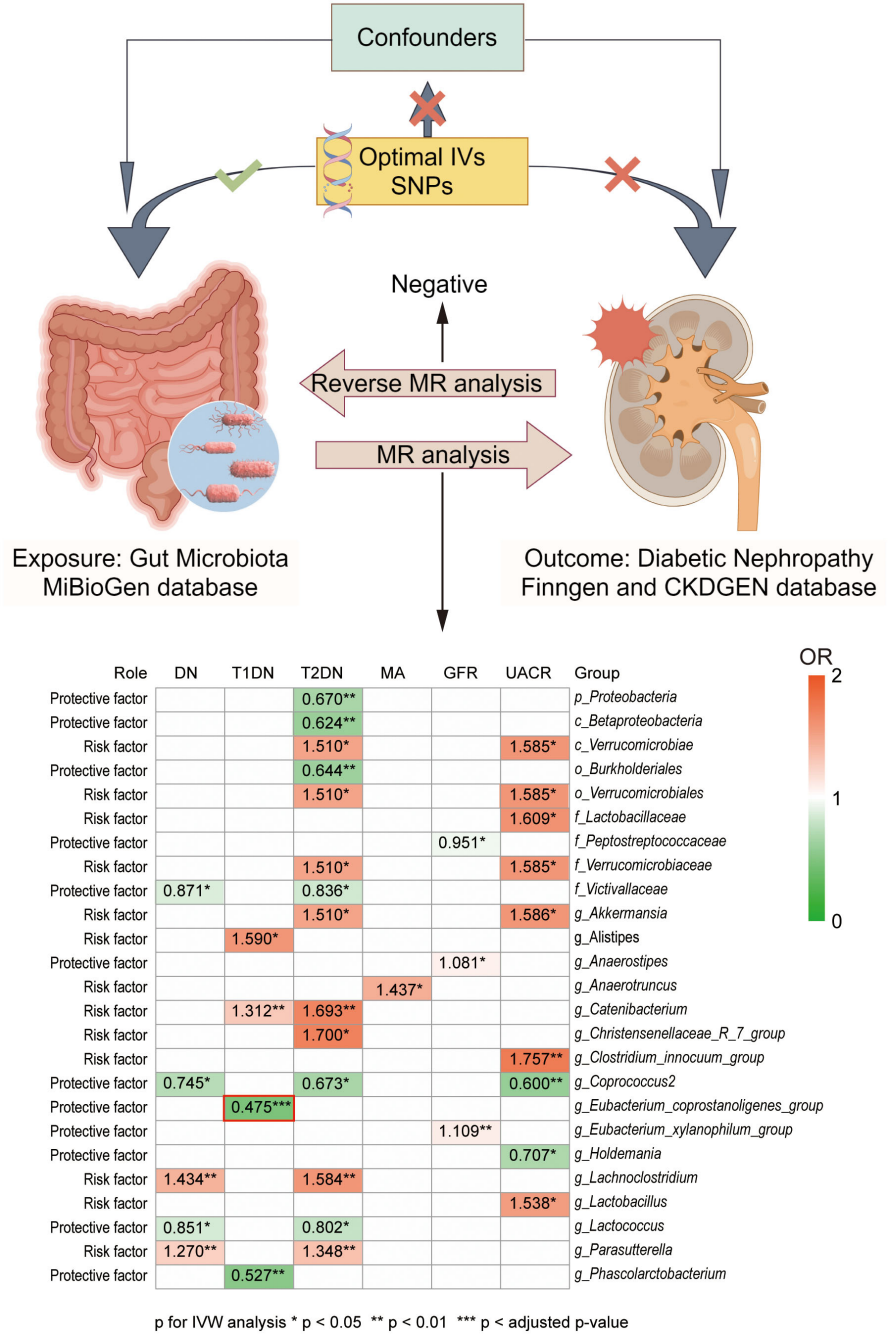
Graphic abstract of present study. Our present study investigated the casual association between GM and DN development at the genetic level. GWAS data of GMs was obtained from the MiBioGen database, while GWAS data of DN were obtained from the FinnGen and CKDGen database. Heat map graph showed the significant causal association between different GM and different outcomes. Red represents an odds ratio (OR) >1, while green represents an OR <1. *p < 0.05, **p < 0.01, ***p < adjusted p-value in inverse variance weighted (IVW) analysis. No significant difference was found in the reverse MR analysis.

In the present study, the causal association between GM and DN was investigated by two-sample MR analysis. Leveraging a substantial amount of GWAS data, we preliminary explored GM’s potential causal influence at the genetic level on the onset and progression of DN. We found that the *g_Eubacterium_coprostanoligenes_group* exhibited a significant negative causal relationship with the development of T1DN. Furthermore, we identified an additional twenty-four GMs of interest with potential causal links to DN. Among these, eleven were identified as protective factors, while thirteen were associated with increased risk. Our study harnessed high-quality GWAS summary data sourced from the MiBioGen consortium, the largest microbiome GWAS consortium, in conjunction with DN GWAS data from the FinnGen and CKDGen consortia. This robust foundation enabled us to derive accurate and reliable results. Considering the substantial global burden of DN and the emerging significance of GM in health and disease, our findings suggest gaining more profound insights into the complex origins of DN and its interaction with GM could be pivotal in making progress toward early diagnosis and preventative strategies for this patient population (**Figure 4** Graphic abstract).

In the present study, most of the identified GM causally associated with DN can be categorized into the taxonomic phyla *p_Bacteroidetes*, *p_Firmicutes*, *p_Proteobacteria* and *p_Verrucomicrobia*. The phyla *Bacteroidetes* and *Firmicutes* are the two largest bacterial phyla, accounting for more than 90% of human GM. Shang J et al. investigated the difference in fecal microbial community between patients with (n=180) and without (n=348) DN in China and reported a higher relative abundance of *p_Verrucomicrobia* but a lower abundance of *p_Firmicutes* in patients with DN, with no change in *p_Bacteroidetes* and *p_Proteobacteria* (20). However, in another study of T2DM patients with chronic kidney disease (CKD), only *p_Verrucomicrobia* was significantly increased compared to healthy controls, and no significant difference was found in the other three identified bacterial phyla (19). To date, no consensus has been reached regarding how GM changes in DN populations (17, 21). Alterations in metabolic regulation, including short-chain fatty acids (SCFAs), bile acids, TMAO, uremic toxins, lipopolysaccharide (LPS), and hydrogen sulfide, have been considered as the primary mechanisms by which the GM contributes to the progression of DN (3). For example, SCFAs are the special nutrients and energy components of the intestinal epithelium. They have been reported to play important roles in maintaining the integrity of the intestinal mucosa, inhibiting local intestinal inflammation, and improving gastrointestinal peristalsis (37). Current evidence suggests that SCFAs are mainly produced by *p_Bacteroidetes* and *p_Firmicutes*, with acetate and propionate mainly produced by *p_Bacteroidetes* and butyrate mainly produced by *p_Firmicutes (* 38). TMAO and LPS have been recognized as risk factors for kidney disease and DM (39, 40).

The *g_Eubacterium_coprostanoligenes_group*, which belongs to the *p_Firmicutes*, plays a crucial role in regulating cholesterol metabolism. To our knowledge, no studies have hitherto investigated the association between the *g_Eubacterium_coprostanoligenes_group* and DN development. In this respect, it has been reported that the relative abundance of *g_Eubacterium_coprostanoligenes_group* was significantly decreased in patients with IgA-related nephropathy (41). In rats with kidney yang deficiency syndrome*, g_Eubacterium_coprostanoligenes_group* showed lower abundance than healthy controls (42). Besides, Banjong D et al. showed a higher relative abundance of *g_Eubacterium_coprostanoligenes_group* in patients with early-stage CKD of unknown etiology than those without CKD and in those with early-stage CKD with underlying disease (43). Research on diabetes-associated cognitive dysfunction (DACD) has found that the abundance of *g_Eubacterium_coprostanoligenes_group* was significantly increased in individuals with DACD in the T2DN cohort compared to those without DACD (44). Indeed, evidence on gut dysbiosis obtained from observational studies is not sufficient to clarify the role of *g_Eubacterium_coprostanoligenes_group* in the development of DN. Our results from MR analysis revealed that *g_Eubacterium_coprostanoligenes_group* can protect against T1DN from a genetic standpoint, although further research is warranted to better understand the underlying mechanisms.

Given the important role of *g_Eubacterium_coprostanoligenes_group* in regulating lipid metabolism, we sought to explain its potential mechanisms in relation to *g_Eubacterium_coprostanoligenes_group*, lipid metabolism, and DN interaction. It has been established that *g_Eubacterium_coprostanoligenes_group* lowers blood lipid levels by converting cholesterol to coprostanol, a substance that cannot be absorbed by the intestine. This intricate process may involve steps, including oxidation to 5-cholesten-3-one, followed by alkene isomerization to 4-cholesten-3-one, conjugate reduction, and ketone reduction (45). Although the potential of *g_Eubacterium_coprostanoligenes_group* to reduce blood lipid levels has been observed, their relationship with blood lipid levels remains inconclusive. As Deng M and Wei W showed that *g_Eubacterium_coprostanoligenes_group* was negatively associated with triglyceride, total cholesterol and low-density lipoprotein cholesterol (LDL-C) (46, 47). However, a positive relationship between the abundance level of *g_Eubacterium_coprostanoligenes_group* and the levels of triglyceride, total cholesterol and LDL-C was reported by Lei S and Xie B (48, 49). We speculate that elevated blood lipids might induce the enrichment of *g_Eubacterium_coprostanoligenes_group*. It induces the reduction of cholesterol absorption in the gut induced by *g_Eubacterium_coprostanoligenes_group* may helps to lower blood lipid levels. Therefore, the relationship between lipid levels and the abundance of *g_Eubacterium_coprostanoligenes_group* may exhibit heterogeneity at different stages of the disease. Obesity and hyperlipidemia have been implicated as risk factors for DN development (50, 51). The accumulation and metabolic disorders of lipids is widely thought to cause oxidative stress, inflammatory reactions, and mitochondrial dysfunction. Consequently, this leads to the destruction of podocytes and glomerular interstitial fibrosis during the development of DN (52–54). A meta-analysis reported that statins (commonly used drugs to control cholesterol during clinical practice) contribute to the prevention and treatment of DN by mitigating albuminuria and urinary albumin excretion rates (55). Based on these findings, we speculate that the renoprotective effect of the *g_Eubacterium_coprostanoligenes_group* may be related to its regulatory effect on lipid metabolism.

Besides the lipid-lowering effects mentioned above, the role of *g_Eubacterium_coprostanoligenes_group* in improving intestinal barrier function and regulating butyric acid should not be overlooked. Bai D et al. reported that the colonization of *Eubacterium_coprostanoligenes* maintained the integrity of colonic tissue structure and a healthy mucus layer, and alleviated inflammation and clinical symptoms in fast food diet-fed mouse models (56). A breakdown of the intestinal barrier can lead to microbial translocation, which may further result in the development of enterogenic infection, systemic inflammatory reaction, and even multiple organ dysfunction syndromes. Diabetes is a risk factor for infections, increasing the risk of urinary tract infections by 3.2 to 4.9 folds (57). In addition to the immunosuppressive effects of high glucose, insufficient blood supply and chronic neurological-vascular injury, increased intestinal permeability and intestinal dysbiosis have been considered as very important factors (58). When comorbid with chronic kidney disease, patients may have a higher risk of urinary tract infections, which are associated with immunocompromise, advanced age, and urinary tract obstruction (59). As common pathogenic bacteria of urinary tract infections, Gram-negative bacteria, including Escherichia coli, Citrobacter spp. and Enterobacter aerogenes, should be given more attention (60). Pyuria is an independent risk factor for progression of kidney disease and may accelerate progression to ESRD (59, 61). A well-functioning intestinal barrier is crucial for preventing DN by inhibiting microbial translocation and the onset of disease. Butyrate, one of the key molecules of SCFAs, is mainly produced by *p_Firmicutes*, including *g_Eubacterium_coprostanoligenes_group (* 38). Although the protective mechanisms of butyrate in DN remain largely unclear, most studies suggest that butyrate can prevent DN progression via inhibition of inflammation, oxidative stress, fibrosis and apoptosis (62, 63). Consistently, it was found that supplementation of exogenous butyrate could restore the composition of GM in db/db mice (64). In addition, supplementation of probiotics, *Bifidobacterium pseudolongum* and *Pediococcus acidilactici FZU106*, alleviated intestinal barrier function and reduced excessive lipid accumulation in the liver by increasing the abundance of the *g_Eubacterium_coprostanoligenes_group (* 65, 66). Decreasing the abundance of *g_Clostridium_innocuum_group*, one of the pathogenic GM identified in present study, is another mechanism underlying the therapeutic effect of *Pediococcus acidilactici FZU106 (* 66). Indeed, the *g_Eubacterium_coprostanoligenes_group* plays a positive role in maintaining human health from multiple perspectives. Overall, more emphasis should be placed on the renoprotective effects of *g_Eubacterium_coprostanoligenes_group* in future studies.

The identified GM species belong to *p_Firmicutes*, most of which are producers of SCFAs, exhibited an inconsistent effect on the development of DN. *G_Lactococcus, g_Phascolarctobacterium*, *g_Anaerostipes* and *g_Coprococcus* were identified as protective factors in the present study. *Lactococcus* is one of the most commonly used probiotic bacteria (67). It has been reported that *g_Lactococcus* could ferment carbohydrates to produce lactic acid, which could be further converted to butyrate by other GM (68, 69). *Lactococcus* has long been considered as a favorable vehicle for drug delivery systems. Oral delivery of staphylococcal nuclease by *Lactococcus lactis* showed a beneficial protective effect on T1DM by inhibiting inflammation and regulating the immune system (70). *Lactococcus lactis* modified to express human proinsulin and human IL-10 showed an active role in the prevention and treatment of T1DM and has a favorable safety profile (71, 72). Previous studies have shown that *g_Phascolarctobacterium and g_Coprococcus* are less abundant in DN patients and animal models (73, 74), while *g_Anaerostipes* exhibited higher abundance in DN patients (18). A previous MR analysis reported the causal association between *g_*Anaerostipes and decreased risk of developing T2DM (75). *G_Coprococcus* has a beneficial effect on the prevention of diabetes, which was positively associated with insulin sensitivity but negatively associated with the presence of dysglycaemia (75). It is widely thought that their protective effect on DN may be closely related to the production of SCFAs (76). Paradoxically, *g_Catenibacterium*, *g_Anaerotruncus*, *g_Christensenellaceae_R_7_group*, *g_Clostridium_innocuum_group*, and *g_Lachnoclostridium* have been documented as the risk factors for DN, although they also contribute to the production of SCFAs (77, 78). A higher abundance of *g_Catenibacterium* and *g_Anaerotruncus* was also found in patients with CKD and end-stage renal disease (ESRD) compared to healthy people (79, 80). In an African American male cohort, higher abundance of *g_Catenibacterium* was observed in patients with higher glycosylated hemoglobin(A1c) (81). This result suggests that *g_Catenibacterium* may influence the serum glucose levels. The abundance of *g_Anaerotruncus* was positively associated with the levels of IL-17, indicating that *g_Anaerotruncus* may promote inflammation (82). Xh Su et al. found a positive correlation between the abundance of *g_Christensenellaceae_R_7_group* and the activation of oxidative stress in rats with DN (83). The adverse effects of *g_Lachnoclostridium* in the development of DN could be explained by dysregulated lipid metabolism. In DN cohorts, *g_Lachnoclostridium* exhibited a negative association with HbA1c levels, and a positive correlation with total cholesterol (TC) and triglyceride (TG) levels (84). In short, the evidence obtained from simple correlational analyses does not provide a complete and precise depiction of the involvement of the mentioned microbiota in DN. Further study is needed for better understanding.

It has been reported that *g_Alistipe* belonging to *p_Bacteroidetes* is associated with an increased risk of DN development. As reported by Chen WH et al., *g_Alistipes* demonstrated a positive association with 24-hour urine output and a negative correlation with serum albumin in individuals with DN (84). As a detrimental factor for DN, *g_Alistipes* contributes to the formation of inflammation and epithelial barrier injury (85), which could lead to the development of DN. Some findings of the present study are inconsistent with the prevailing opinion on GM. For instance, while *g_Akkermansia* has been associated with various beneficial effects in previous studies (86, 87), our research identified it as a risk factor. Conversely, *p_Proteobacteria* exhibited the opposite relationship in our study compared to prior findings (88). This discrepancy might attribute to the small sample size of our GWAS data, emphasizing the need for more research.

Our study boasts several strengths in its design. Indeed, using MR analysis to investigate the potential causal association between GM and DN can minimize the impact of confounders. The utilization of GWAS data sourced from well-established databases such as MiBioGen, FinnGen R9, and CKDGen enhances the robustness of our MR analysis. In addition, directionally consistent results were required to obtain robust results, and Bonferroni adjusted p-value was used. Although MR analysis helps to eliminate the influence of confounding factors, the results may also be influenced by pleiotropy and heterogeneity. To address this concern, we conducted several sensitivity analyses, encompassing MR-Egger regression, MR-PRESSO, and Cochran’s Q tests, in an effort to mitigate potential pleiotropic and heterogeneity effects.

Nonetheless, some inevitable limitations should be considered in interpreting our findings. Firstly, non-linear correlations cannot be analyzed because we used aggregated GWAS data from public databases rather than raw data. Secondly, our findings were based on data from individuals of European ancestry and cannot be generalized to other ethnicities, such as African and Asian populations, due to the inherent genetic differences between different ethnicities. Thirdly, further longitudinal studies are needed to confirm the causal association though convincing causal associations were found in our present studies. Fourth, we provided a novel idea of the causal associations between GM and DN development but were not able to differentiate the degree of association based on the different stages of DN. The relationship between disease severity and GM is crucial. It is highly conceivable that different degrees of DN may have different effects on GM, and the abundance of GM may also be influenced by different treatments given to patients with different degrees of DN (18). Therefore, the generalizability of the results may be limited. Fifthly, although we used by far the largest GWAS data of GM, consisting with 14,306 samples, the sample size was small. The sample size of the outcome GWAS data from the CKDGen database was not big enough. Future research would benefit from higher-quality GWAS datasets with larger sample sizes to enhance predictive power and reduce potential errors.

Our study carries important implications for further research and clinical practice in the management of DN. Further basic research should focus on unraveling the mechanisms responsible for the regulatory effects of the identified GM on DN development, especially the *g_Eubacterium_coprostanoligenes_group*. In addition, high-quality longitudinal clinical studies are needed to provide more conclusive causal evidence. In clinical practice, our research underscores the pivotal role of GM as part of a comprehensive approach to early DN diagnosis and treatment. So far, dietary interventions and exposure to probiotics and prebiotics have been proven effective in modulating GM distribution and function. Fecal microbiota transplantation (FMT) stands as an emerging therapeutic method for adjusting GM distribution and improving the intestinal environment, with its beneficial effects and safety well-documented in preclinical studies for diabetes and DN (89, 90). With further clinical research, FMT is poised to become a crucial tool in DN treatment.

## 5 Conclusion

Our present study provides novel insights into the association between GM and DN. The identified GM may be potential diagnostic biomarkers and therapeutic targets for DN. However, these causal associations and the potential mechanism warrant further investigation in the future. Our efforts may contribute to the development of innovative strategies for the early diagnosis and treatment of DN with emphasis on GM.

## Data availability statement

The original contributions presented in the study are included in the article/**Supplementary Material**. Further inquiries can be directed to the corresponding author.

## Author contributions

YF: Writing – original draft, Software, Project administration, Methodology, Formal Analysis, Data curation, Conceptualization. YZ: Writing – original draft, Visualization, Software, Methodology, Formal Analysis, Data curation. QL: Writing – review & editing, Supervision, Funding acquisition. ZZ: Writing – review & editing. CR: Writing – review & editing. XZ: Writing – review & editing, Supervision, Project administration, Funding acquisition, Conceptualization.
