# Supplementary material for: Assessing the causal relationship between gut microbiota and diabetic nephropathy: insights from two-sample Mendelian randomization
Source: Front Endocrinol (Lausanne). 2024 Mar 18;15:1329954. doi: 10.3389/fendo.2024.1329954 (PMC10982433; doi:10.3389/fendo.2024.1329954)
Supplement: Supplementary file 1 [file DataSheet_1.pdf]

Assessing the Causal Relationship between Gut Microbiota and Diabetic Nephropathy: Insights from Two-Sample Mendelian Randomization

Supplemental file 1 The visualisation results for describing the causal association between all identified gut microbiota and the risk of developing diabetic nephropathy.

Figure List

Part One all identified gut microbiota and diabetic nephropathy (DN)

family.Victivallaceae......1
genus.Catenibacterium......1
genus.Coprococcus2......2
genus.Lachnoclostridium......2
genus.Lactococcus......3
genus.Parasutterella......3

Part Two all identified gut microbiota and type 1 diabetic nephropathy (T1DN)

genus.Alistipes......4
genus.Catenibacterium......4
genus.Christensenellaceae_R_7_group......5
genus.Eubacterium_coprostanoligenes_group......5
genus.Phascolarctobacterium......6

Part Three all identified gut microbiota and type 2 diabetic nephropathy (T2DN)

phylum.Proteobacteria......7
class.Betaproteobacteria......7
class.Verrucomicrobiae......8
order.Burkholderiales......8
order.Verrucomicrobiales......9
family.Verrucomicrobiaceae......9
family.Victivallaceae......10
genus.Akkermansia......10
genus.Coprococcus2......11
genus.Lachnoclostridium......11
genus.Lactococcus......12
genus.Parasutterella......12

Part Four all identified gut microbiota and glomerular filtration rate (GFR)

family.Peptostreptococcaceae......13

genus.Anaerostipes..13
genus.Eubacterium_xylanophilum_group..14

Part Five all identified gut microbiota and microalbuminuria (MA)

genus.Anaerotruncus..15

Part Six all identified gut microbiota and urinary albumin to creatinine ratio(UACR)

class.Verrucomicrobiae...16
order.Verrucomicrobiales...16
family.Verrucomicrobiaceae..17
genus.Akkermansia..17
genus.Clostridium_innocuum_group..18
genus.Coprococcus2..18
genus.Holdemania...19
genus.Lactobacillus..19

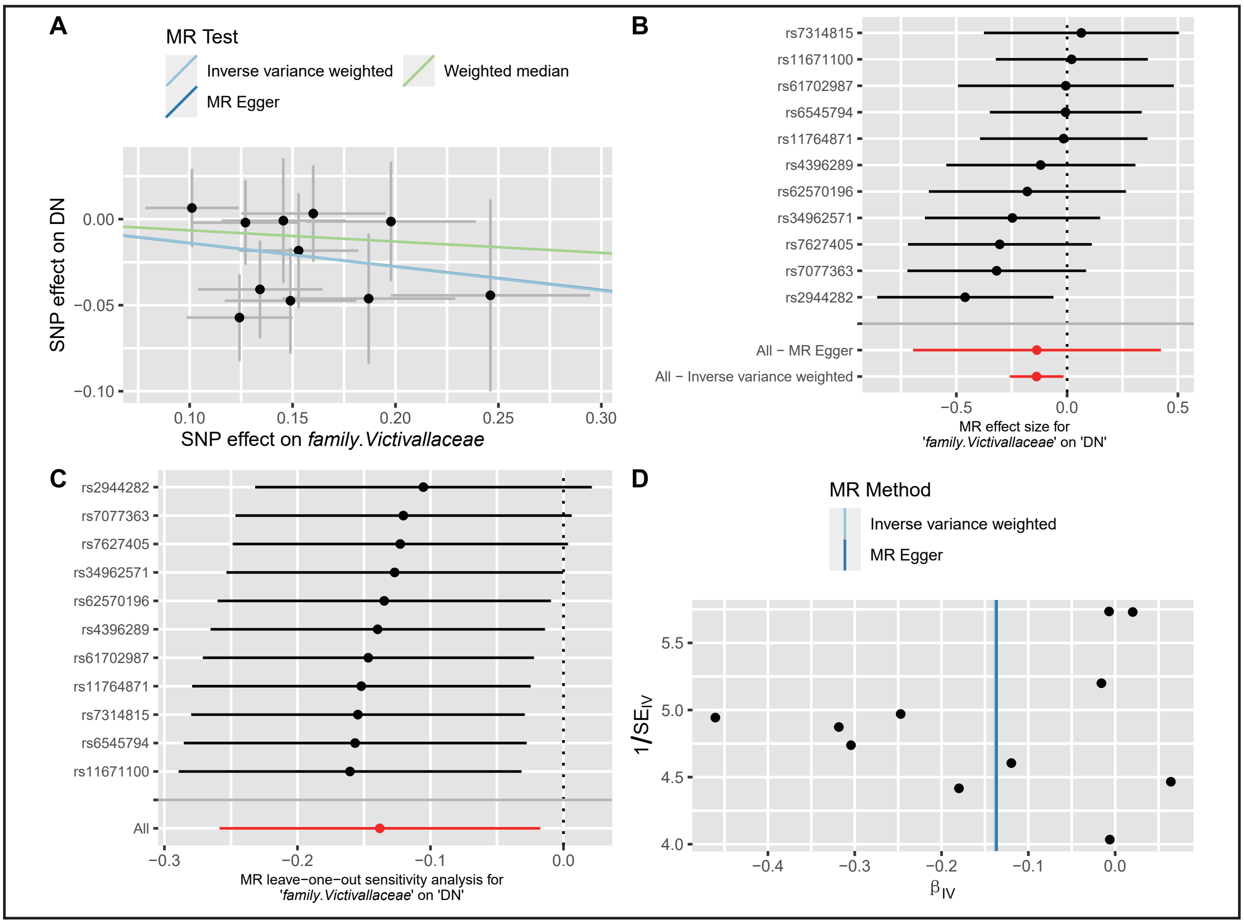

Figure S1 The visualisation results for describing the causal association between ***f_Victivallaceae*** and diabetic nephropathy. (A) Scatter plot; (B) Forest plot; (C) Leave-one-out sensitivity analysis plot; (D) Funnel plot.

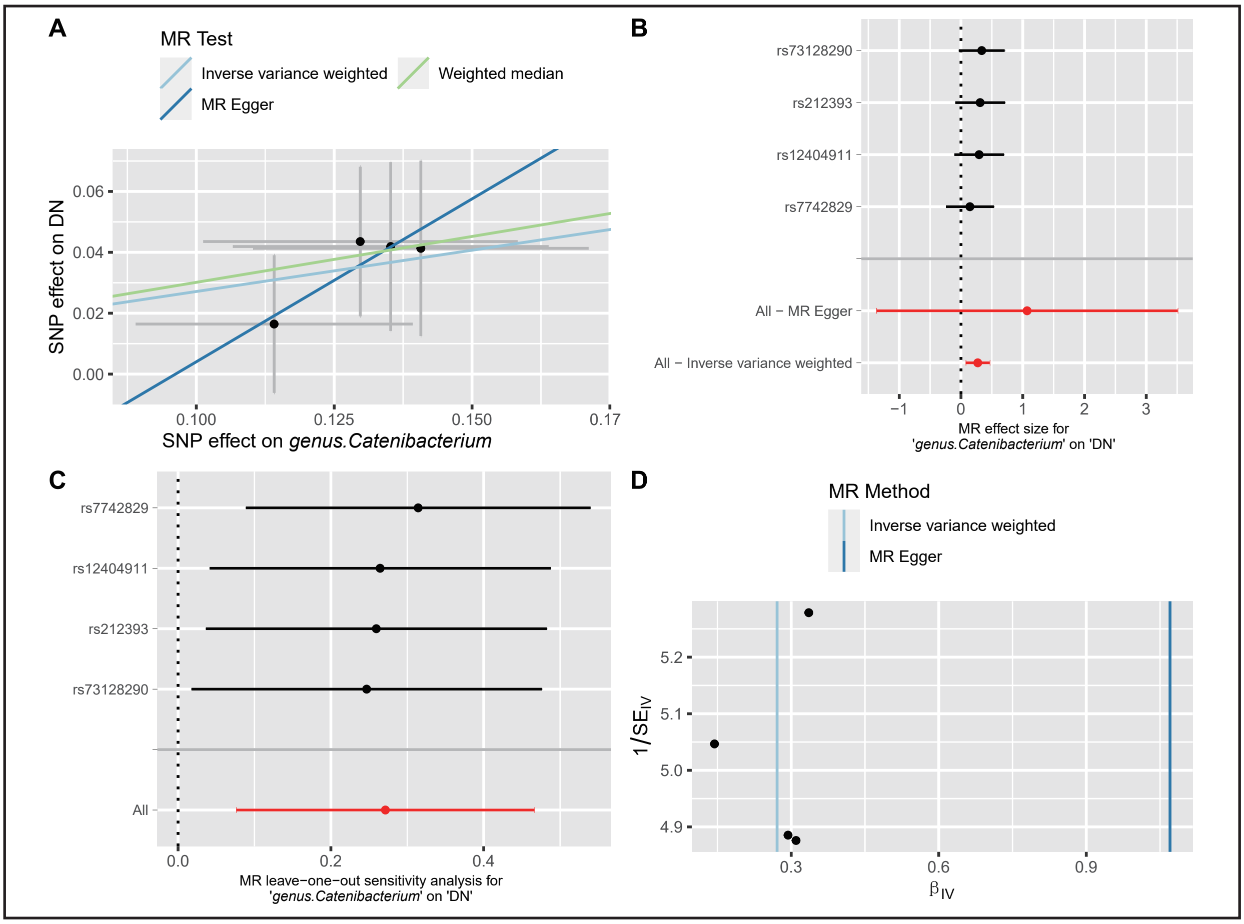

Figure S2 The visualisation results for describing the causal association between ***g_Catenibacterium*** and diabetic nephropathy. (A) Scatter plot; (B) Forest plot; (C) Leave-one-out sensitivity analysis plot; (D) Funnel plot.

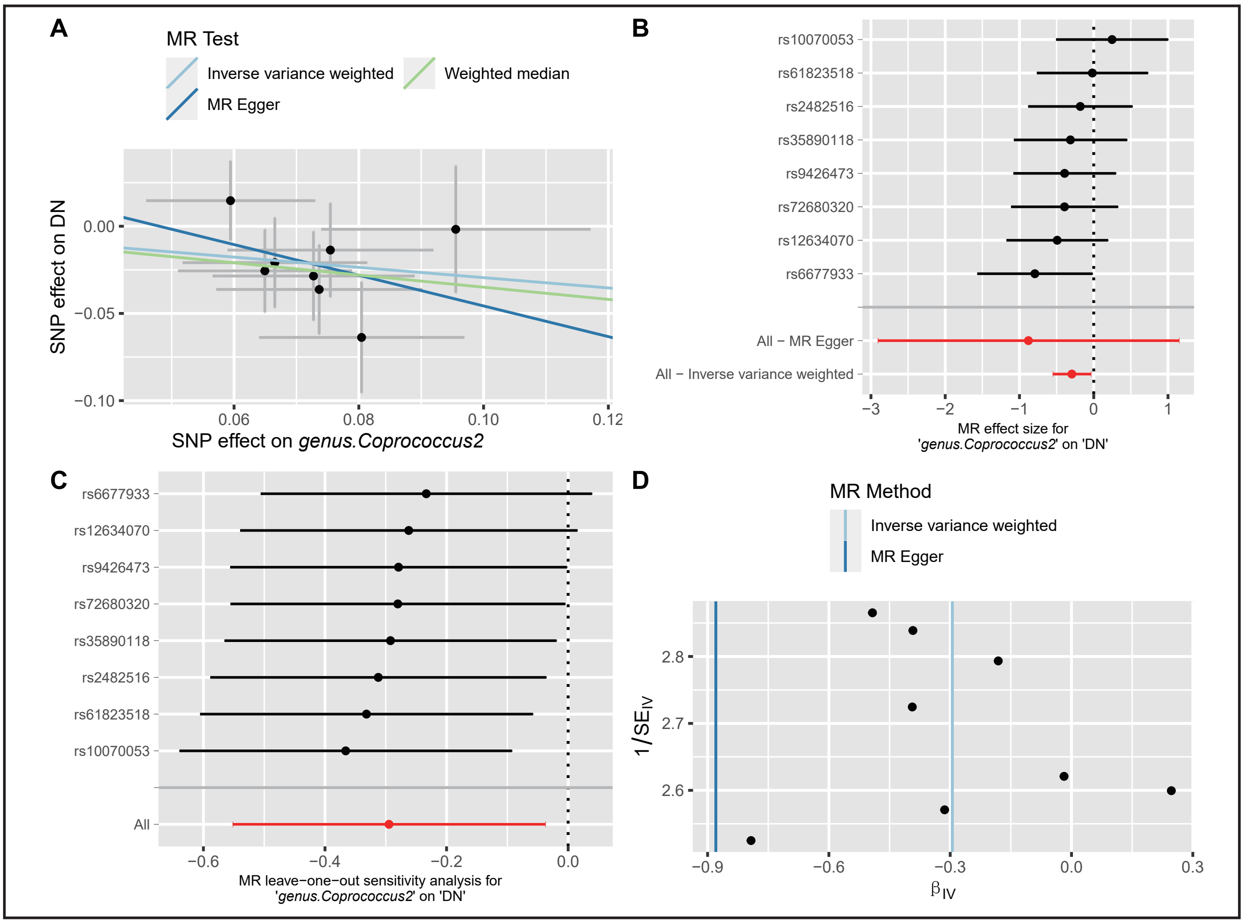

Figure S3 The visualisation results for describing the causal association between ***g_Coprococcus2*** and diabetic nephropathy. (A) Scatter plot; (B) Forest plot; (C) Leave-one-out sensitivity analysis plot; (D) Funnel plot.

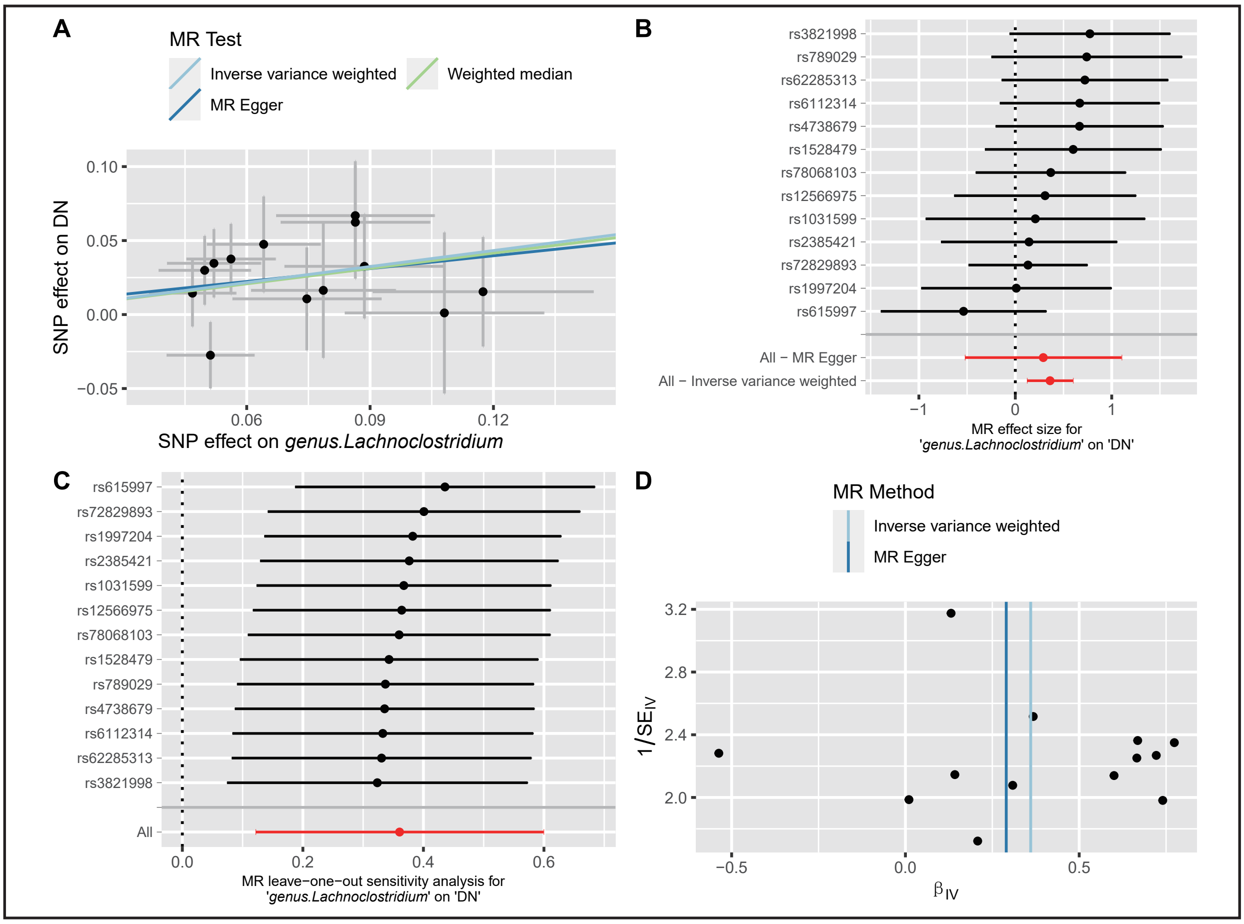

Figure S4 The visualisation results for describing the causal association between ***g_Lachnoclostridium*** and diabetic nephropathy. (A) Scatter plot; (B) Forest plot; (C) Leave-one-out sensitivity analysis plot; (D) Funnel plot.

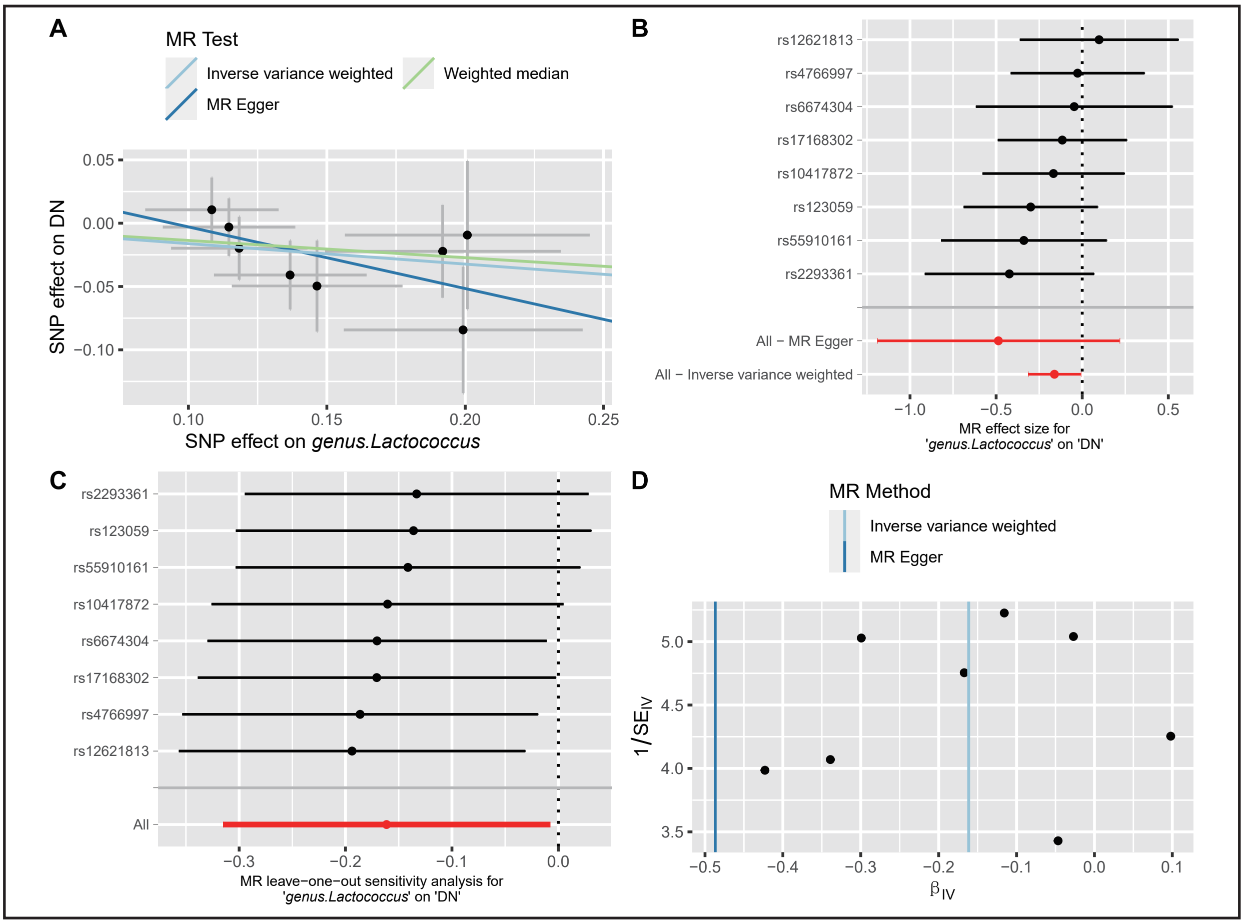

Figure S5 The visualisation results for describing the causal association between ***g_Lactococcus*** and diabetic nephropathy. (A) Scatter plot; (B) Forest plot; (C) Leave-one-out sensitivity analysis plot; (D) Funnel plot.

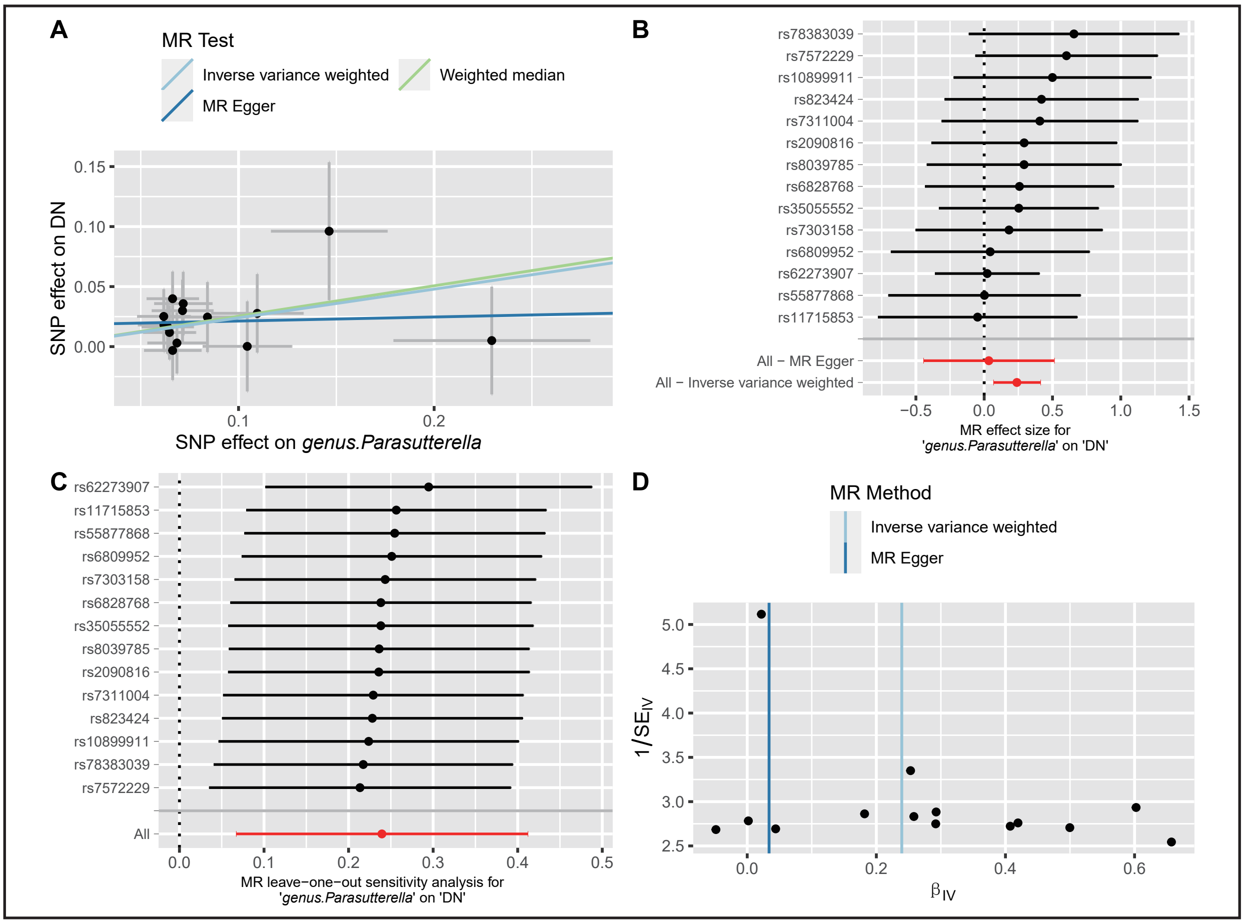

Figure S6 The visualisation results for describing the causal association between ***g_Parasutterella*** and diabetic nephropathy. (A) Scatter plot; (B) Forest plot; (C) Leave-one-out sensitivity analysis plot; (D) Funnel plot.

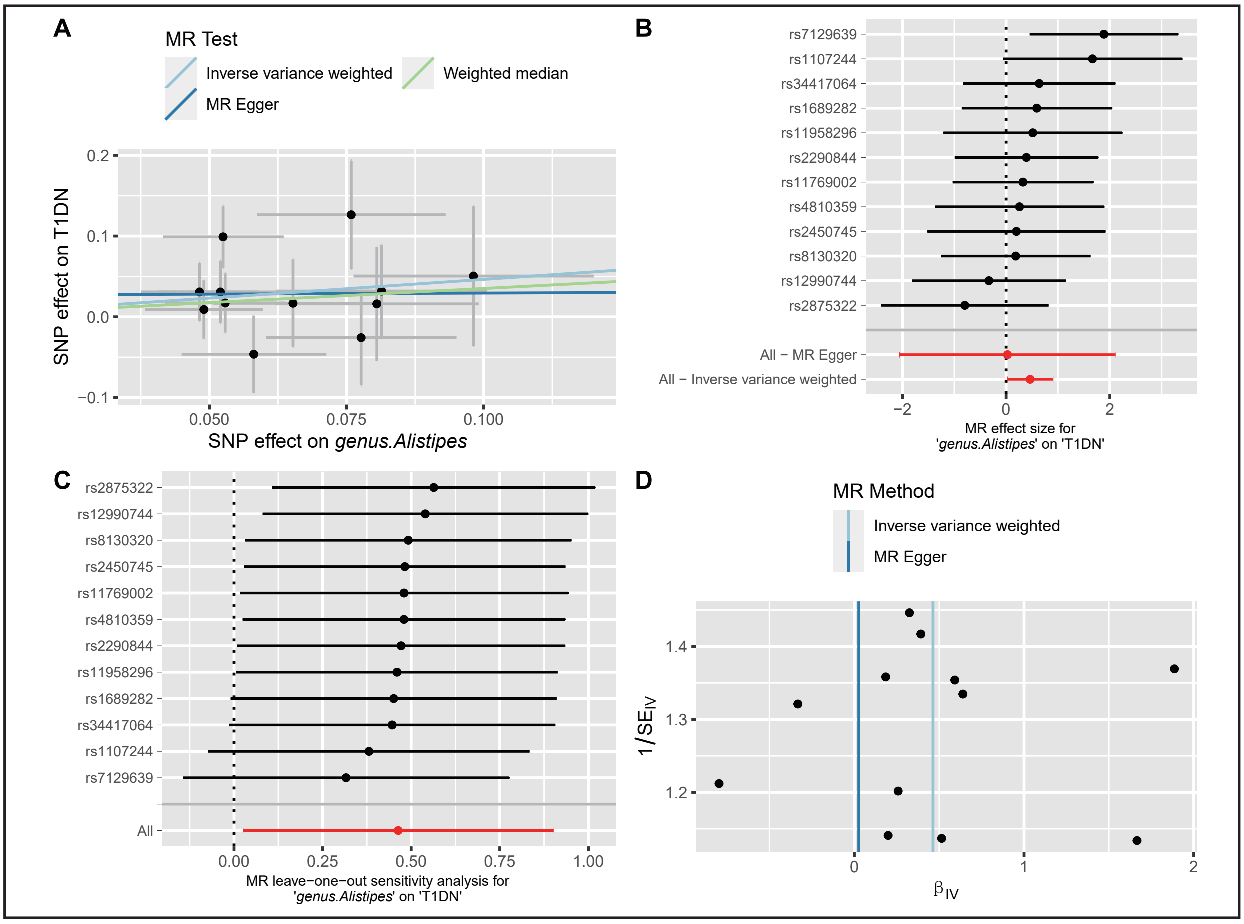

Figure S7 The visualisation results for describing the causal association between ***g_Alistipes*** and type 1 diabetic nephropathy. (A) Scatter plot; (B) Forest plot; (C) Leave-one-out sensitivity analysis plot; (D) Funnel plot.

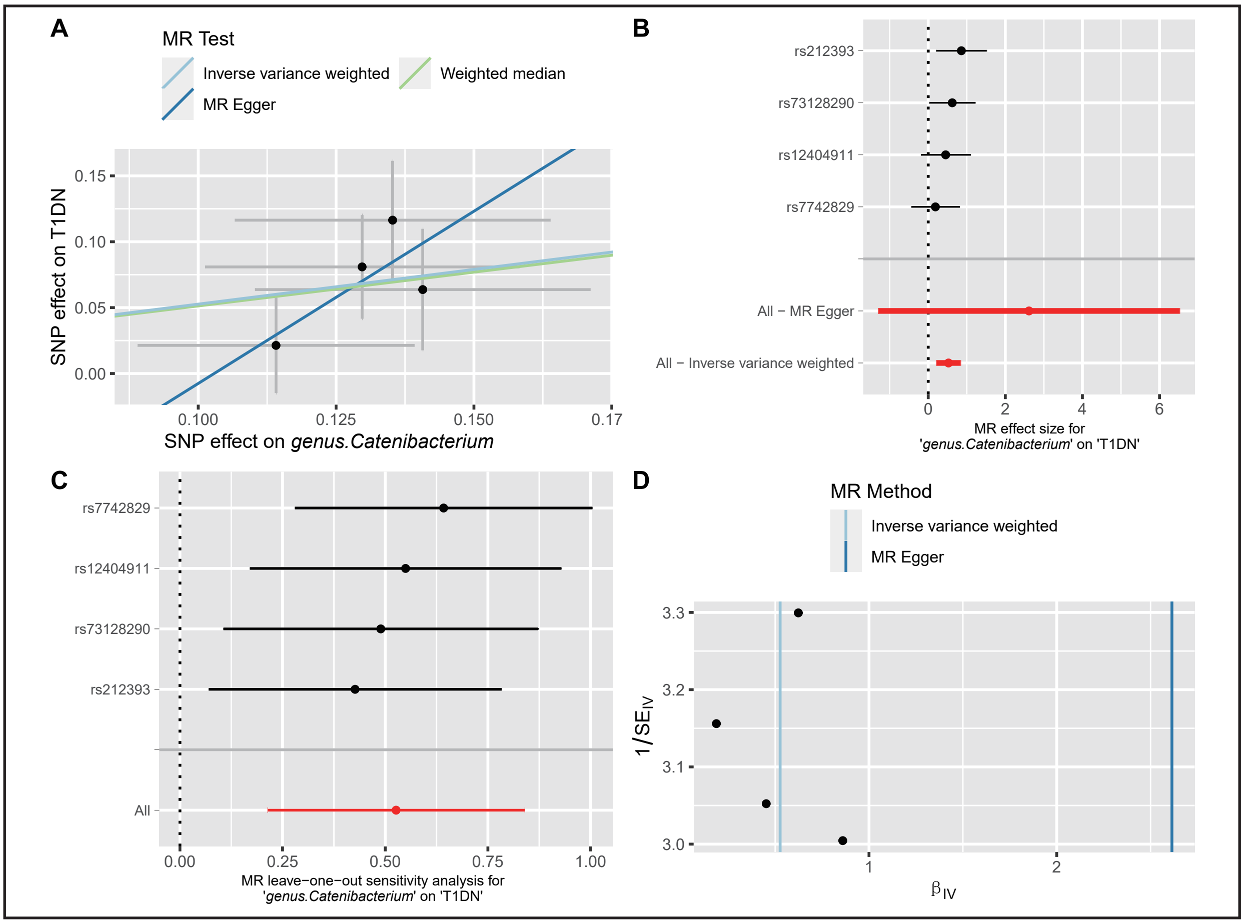

Figure S8 The visualisation results for describing the causal association between ***g_Catenibacterium*** and type 1 diabetic nephropathy. (A) Scatter plot; (B) Forest plot; (C) Leave-one-out sensitivity analysis plot; (D) Funnel plot.

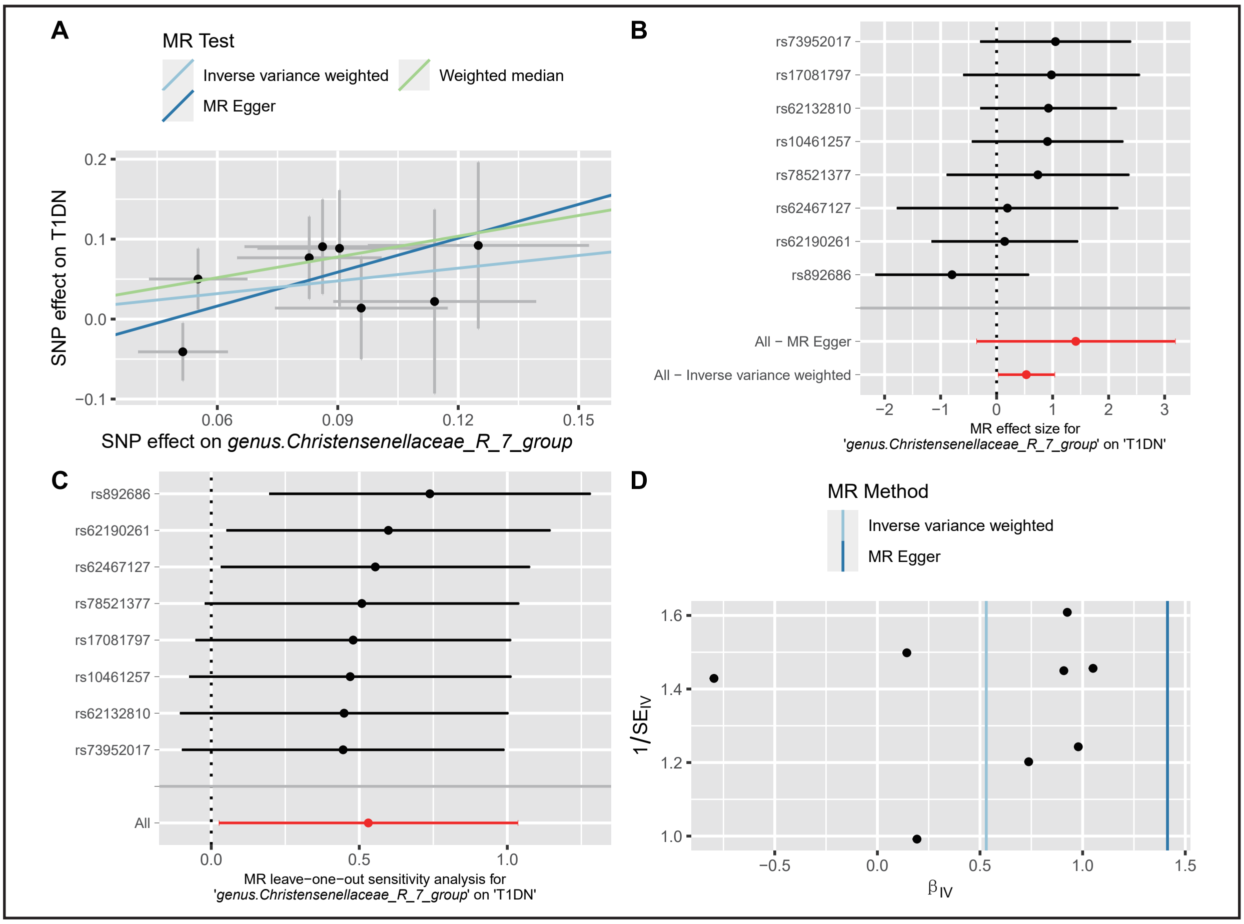

Figure S9 The visualisation results for describing the causal association between ***g_Christensenellaceae_R_7_group*** and type 1 diabetic nephropathy. (A) Scatter plot; (B) Forest plot; (C) Leave-one-out sensitivity analysis plot; (D) Funnel plot.

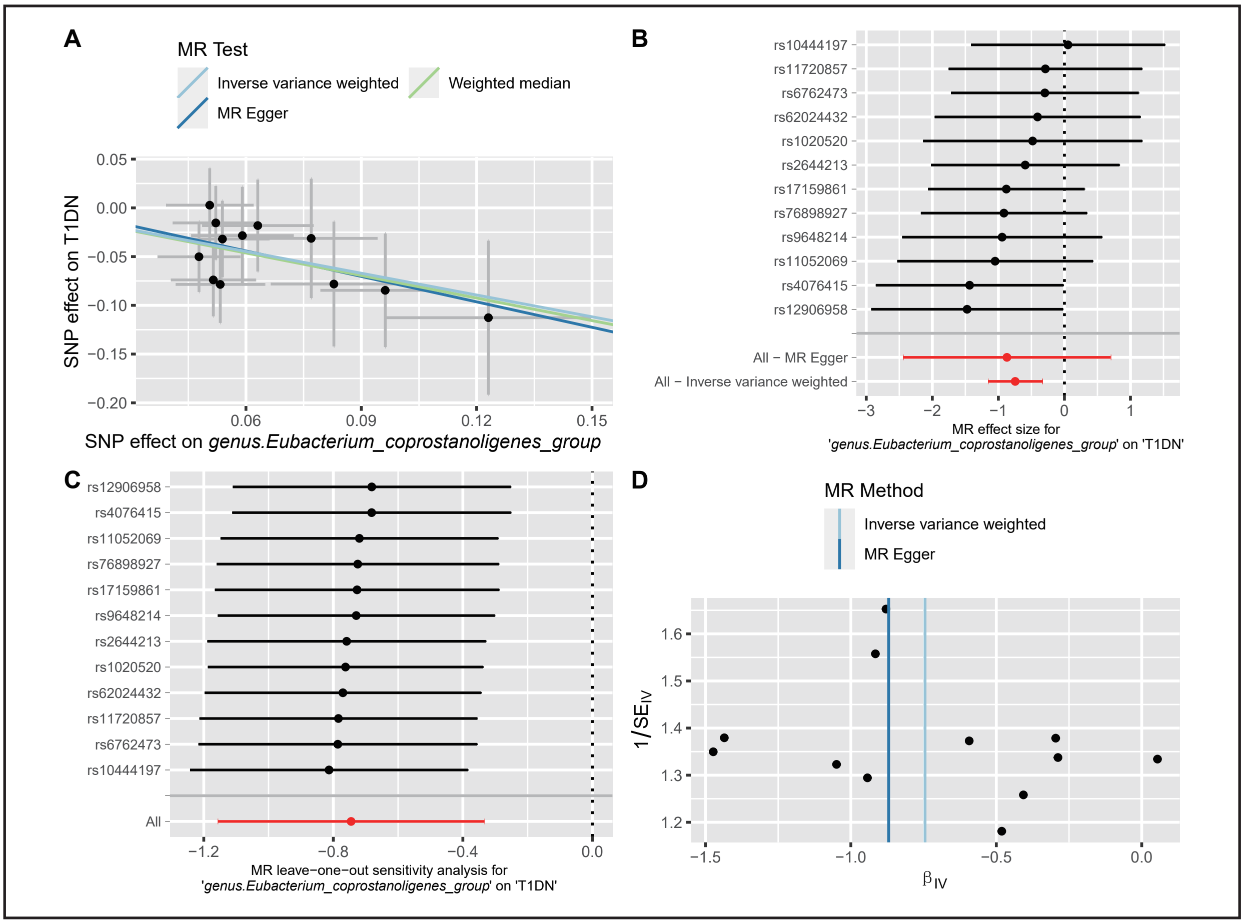

Figure S10 The visualisation results for describing the causal association between ***g_Eubacterium_coprostanoligenes_group*** and type 1 diabetic nephropathy. (A) Scatter plot; (B) Forest plot; (C) Leave-one-out sensitivity analysis plot; (D) Funnel plot.

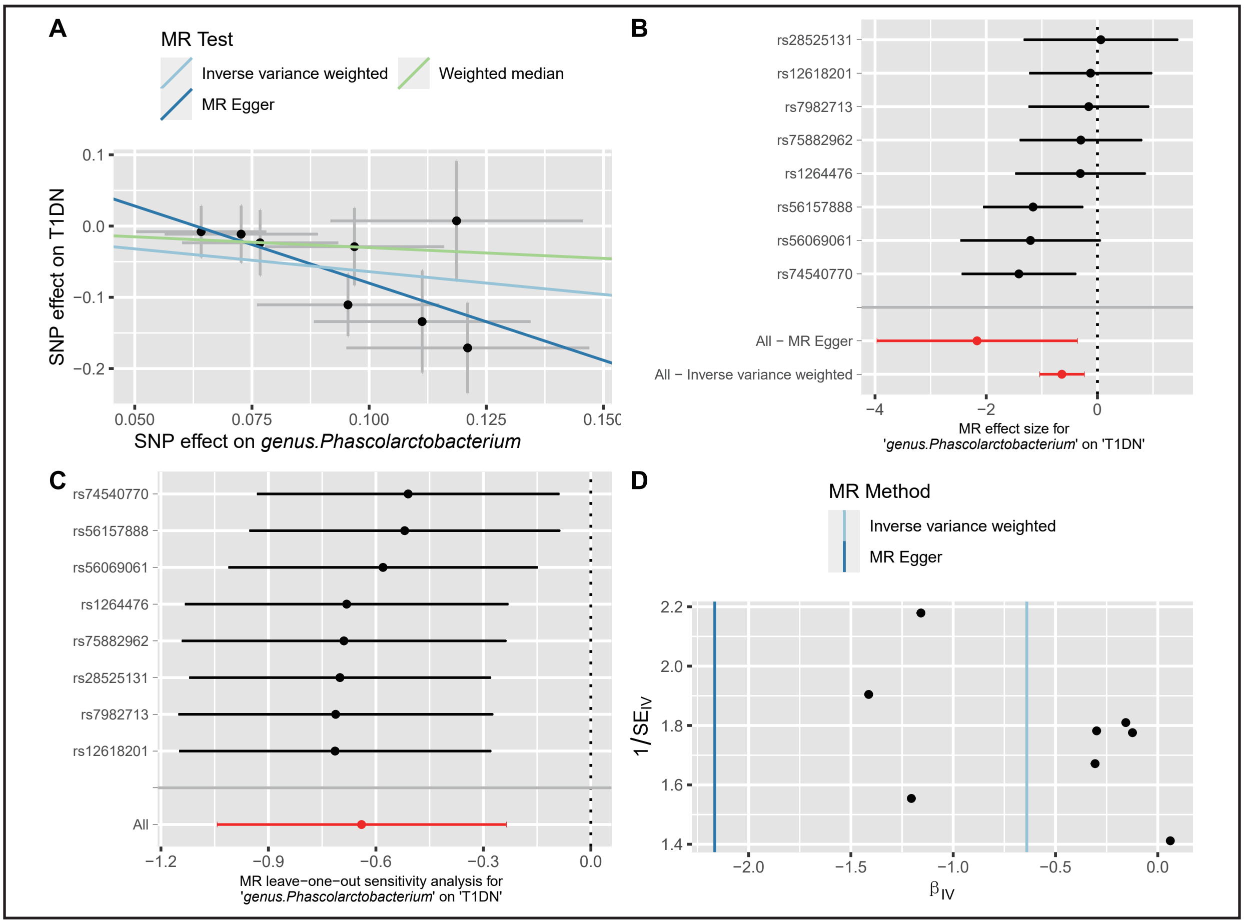

Figure S11 The visualisation results for describing the causal association between ***g_Phascolarctobacterium*** and type 1 'diabetic nephropathy. (A) Scatter plot; (B) Forest plot; (C) Leave-one-out sensitivity analysis plot; (D) Funnel plot.

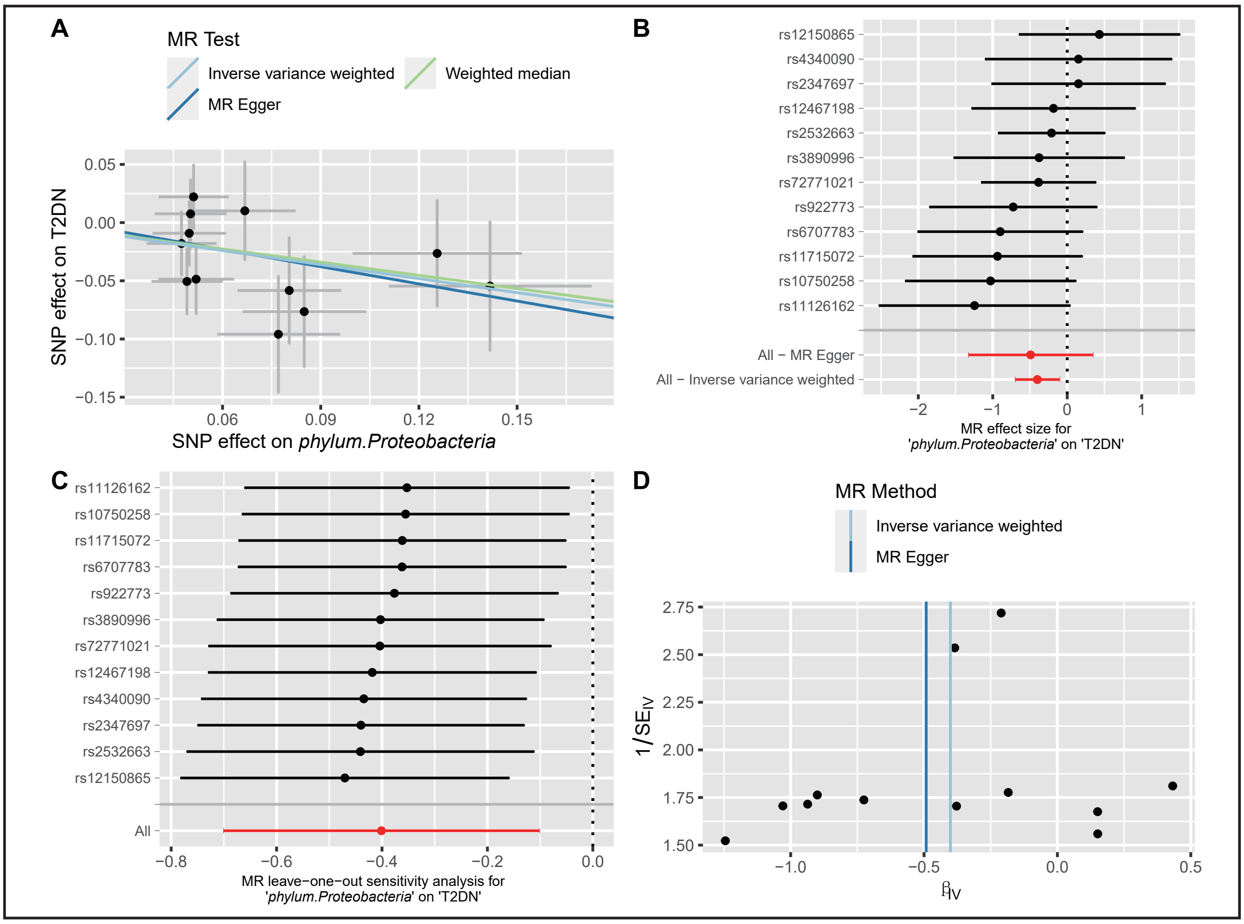

Figure S12 The visualisation results for describing the causal association between ***p_Proteobacteria*** and type 2 diabetic nephropathy. (A) Scatter plot; (B) Forest plot; (C) Leave-one-out sensitivity analysis plot; (D) Funnel plot.

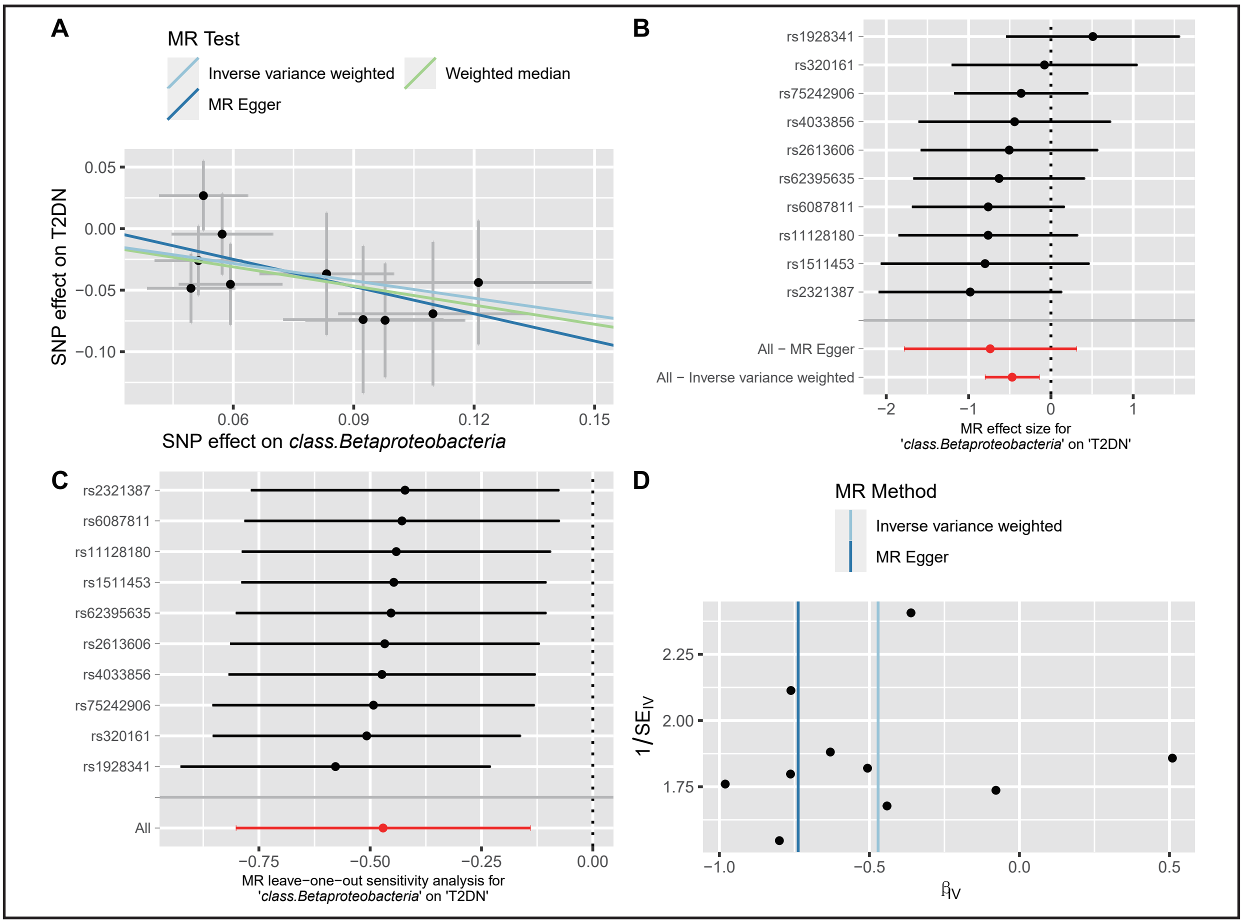

Figure S13 The visualisation results for describing the causal association between ***c_Betaproteobacteria*** and type 2 diabetic nephropathy. (A) Scatter plot; (B) Forest plot; (C) Leave-one-out sensitivity analysis plot; (D) Funnel plot.

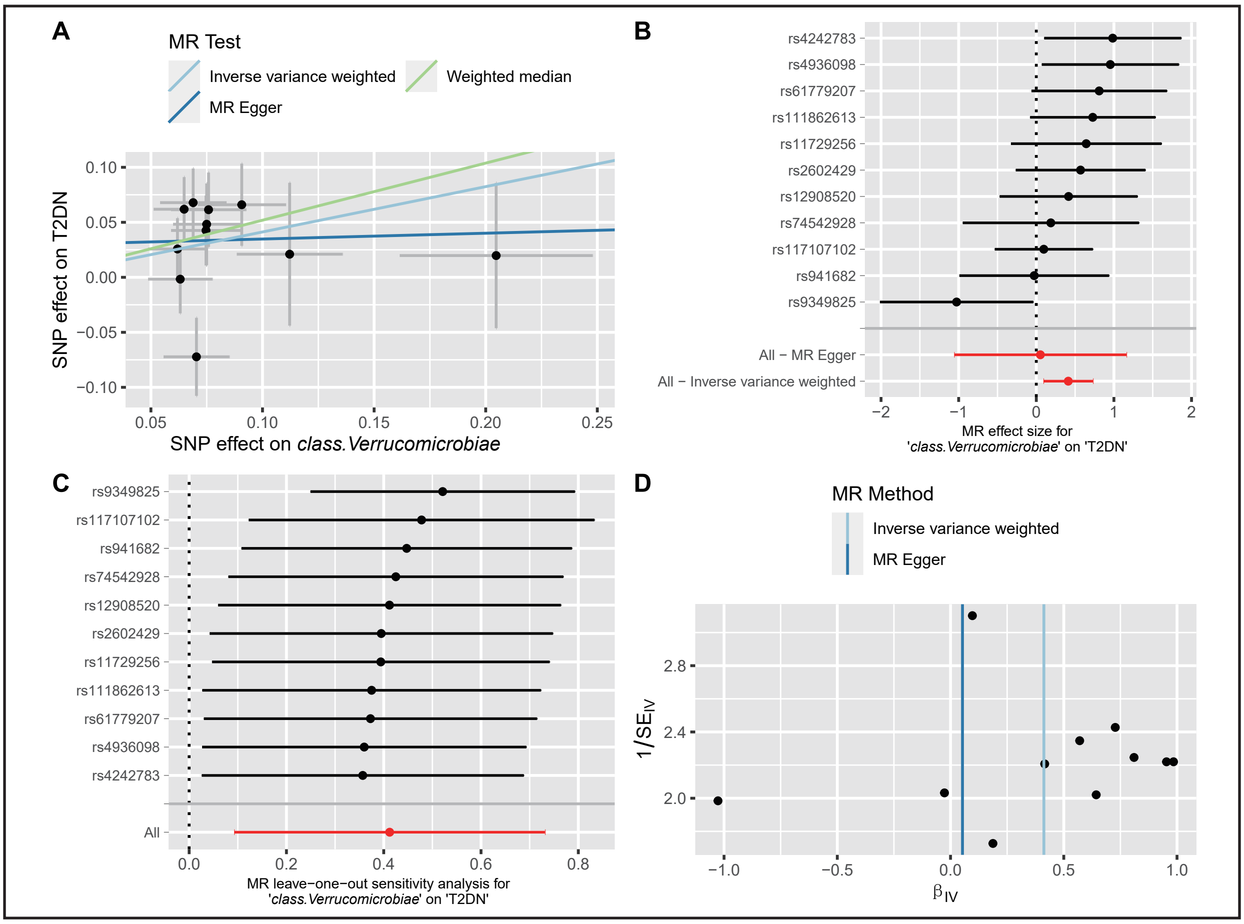

Figure S14 The visualisation results for describing the causal association between ***c_Verrucomicrobiae*** and type 2 diabetic nephropathy. (A) Scatter plot; (B) Forest plot; (C) Leave-one-out sensitivity analysis plot; (D) Funnel plot.

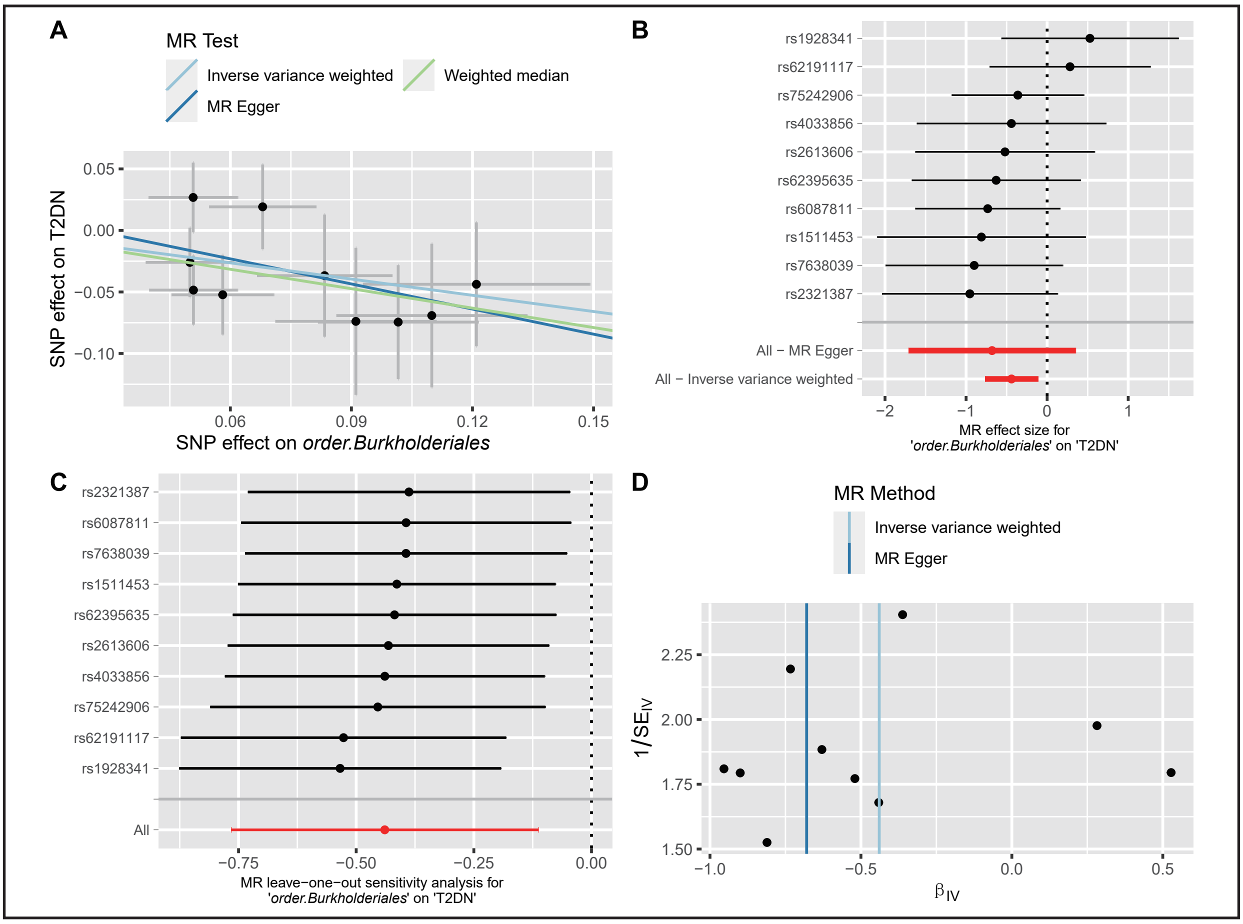

Figure S15 The visualisation results for describing the causal association between ***o_Burkholderiales*** and type 2 diabetic nephropathy. (A) Scatter plot; (B) Forest plot; (C) Leave-one-out sensitivity analysis plot; (D) Funnel plot.

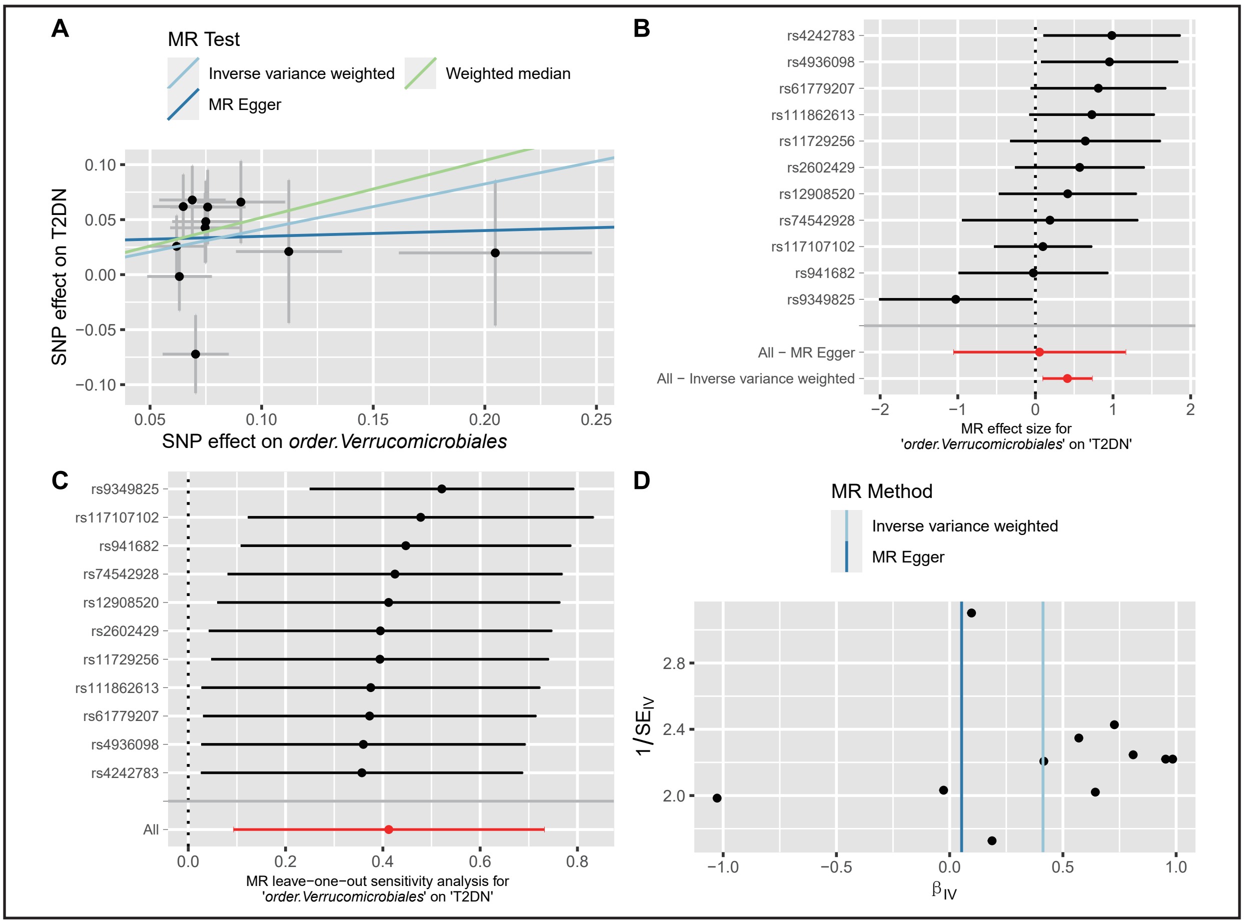

Figure S16 The visualisation results for describing the causal association between ***o_Verrucomicrobiales*** and type 2 diabetic nephropathy. (A) Scatter plot; (B) Forest plot; (C) Leave-one-out sensitivity analysis plot; (D) Funnel plot.

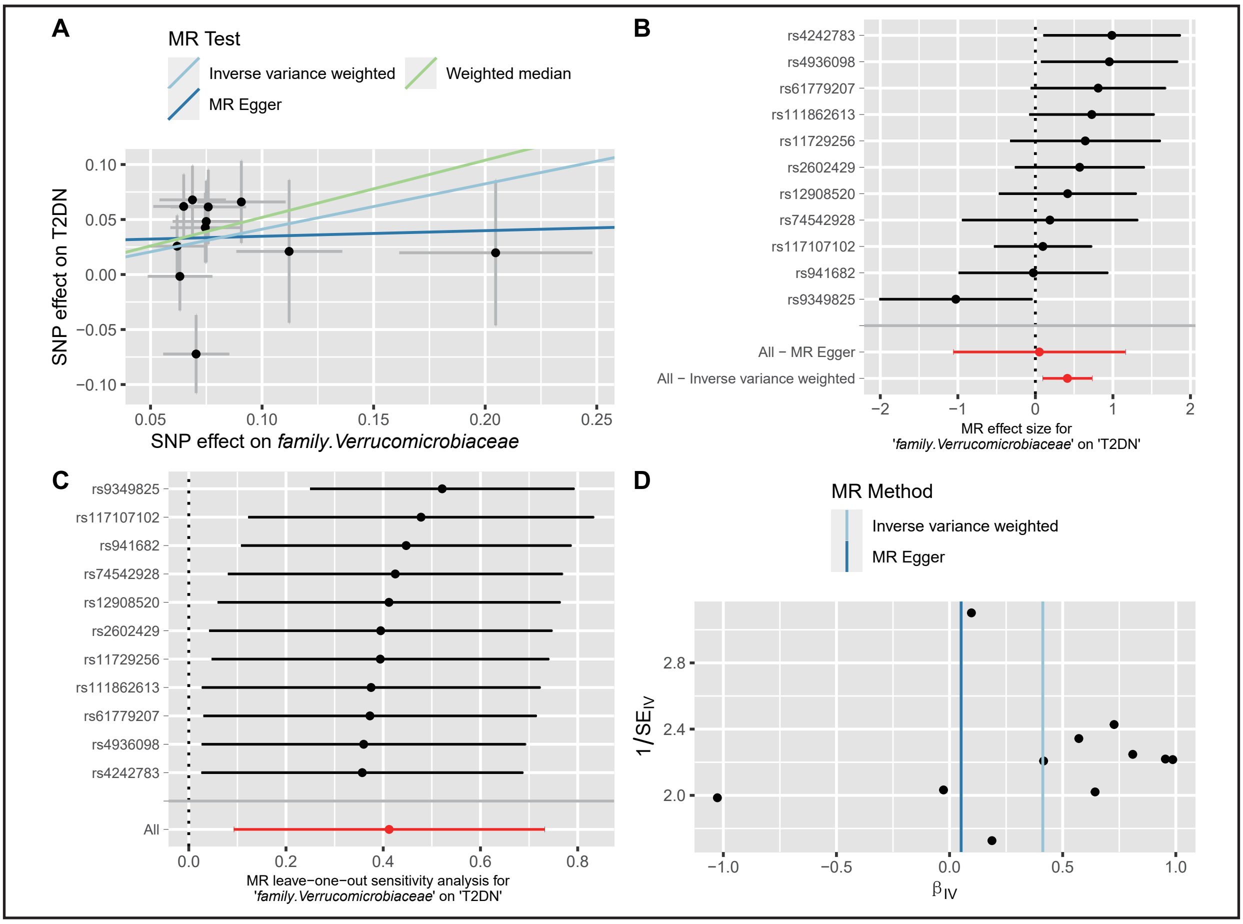

Figure S17 The visualisation results for describing the causal association between ***f_Verrucomicrobiaceae*** and type 2 diabetic nephropathy. (A) Scatter plot; (B) Forest plot; (C) Leave-one-out sensitivity analysis plot; (D) Funnel plot.

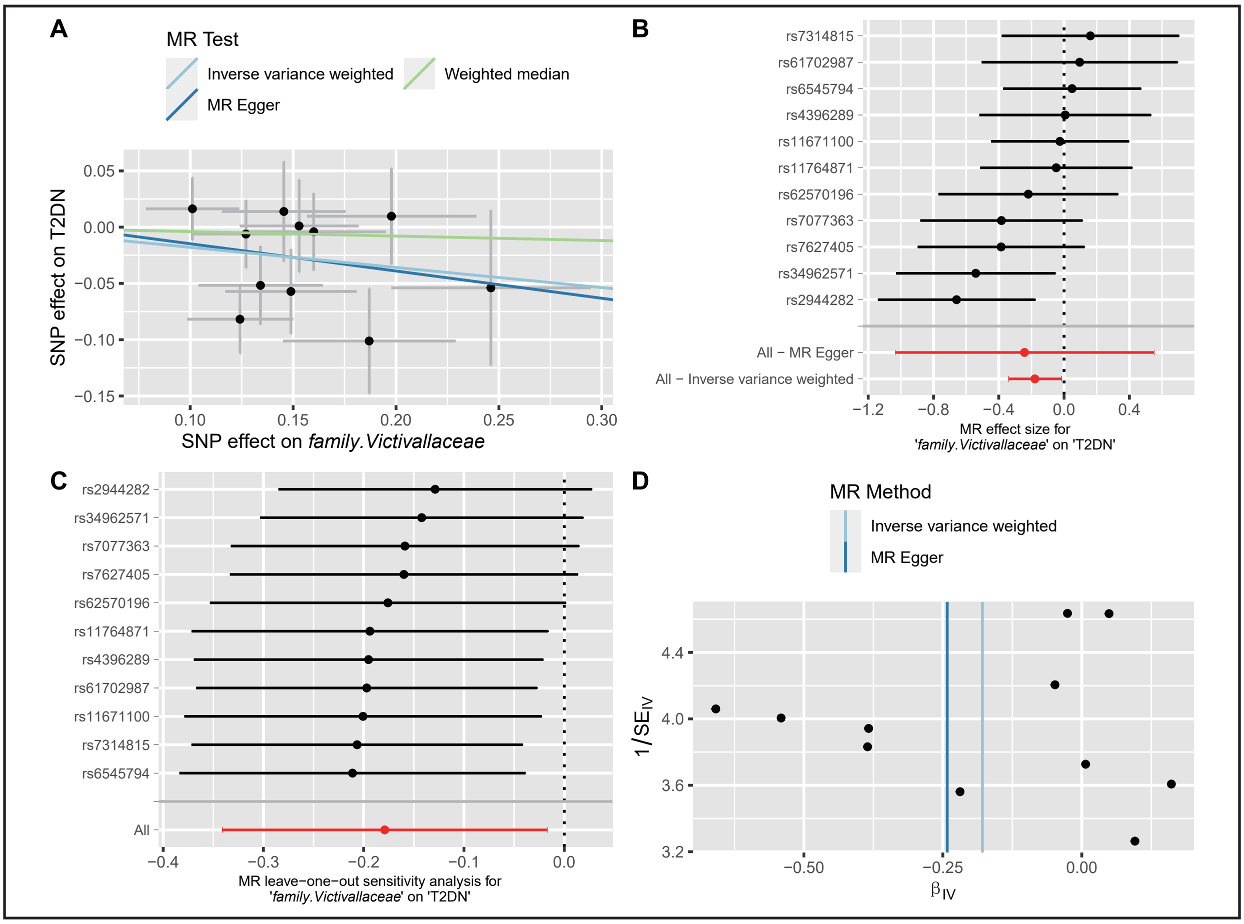

Figure S18 The visualisation results for describing the causal association between ***f_Victivallaceae*** and type 2 diabetic nephropathy. (A) Scatter plot; (B) Forest plot; (C) Leave-one-out sensitivity analysis plot; (D) Funnel plot.

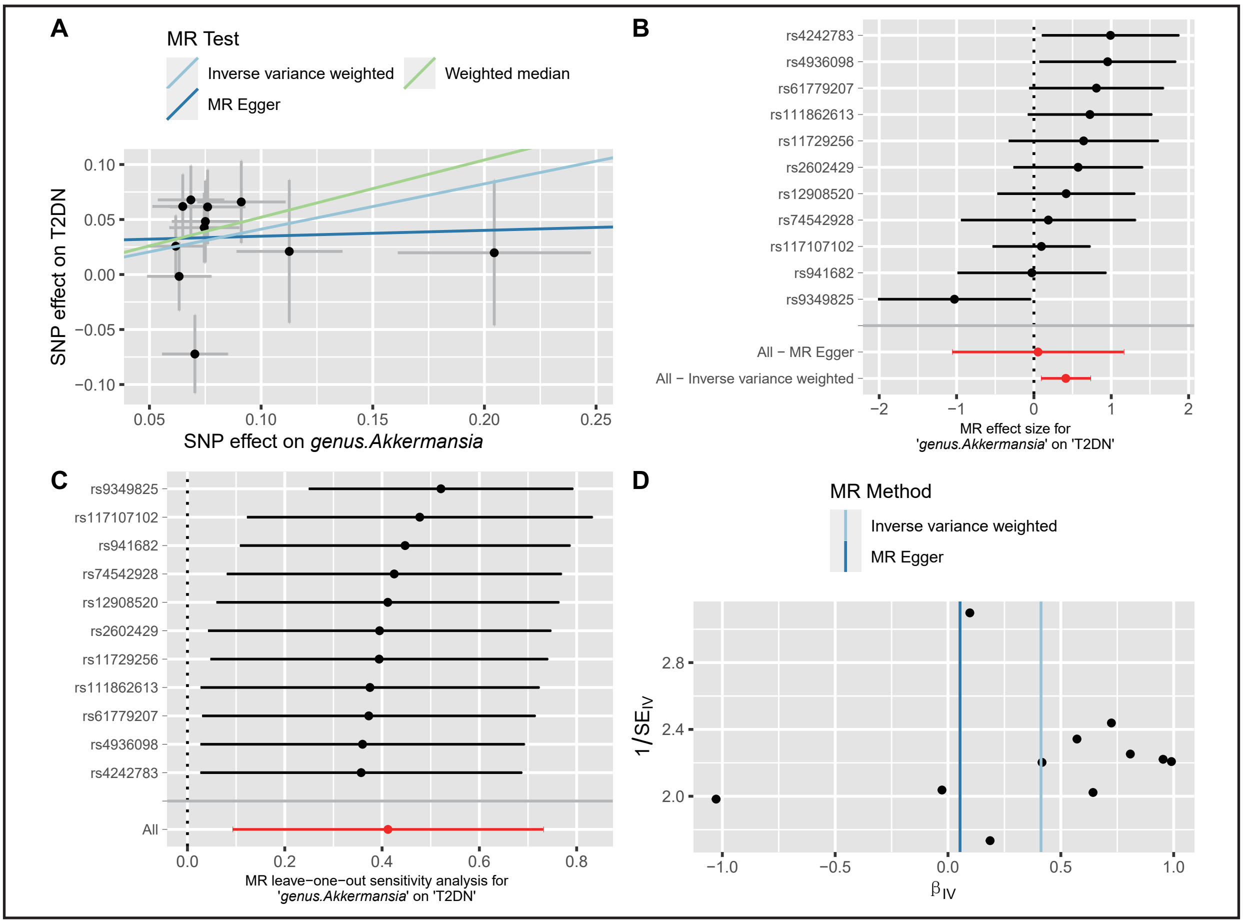

Figure S19 The visualisation results for describing the causal association between ***g_Akkermansia*** and type 2 diabetic nephropathy. (A) Scatter plot; (B) Forest plot; (C) Leave-one-out sensitivity analysis plot; (D) Funnel plot.

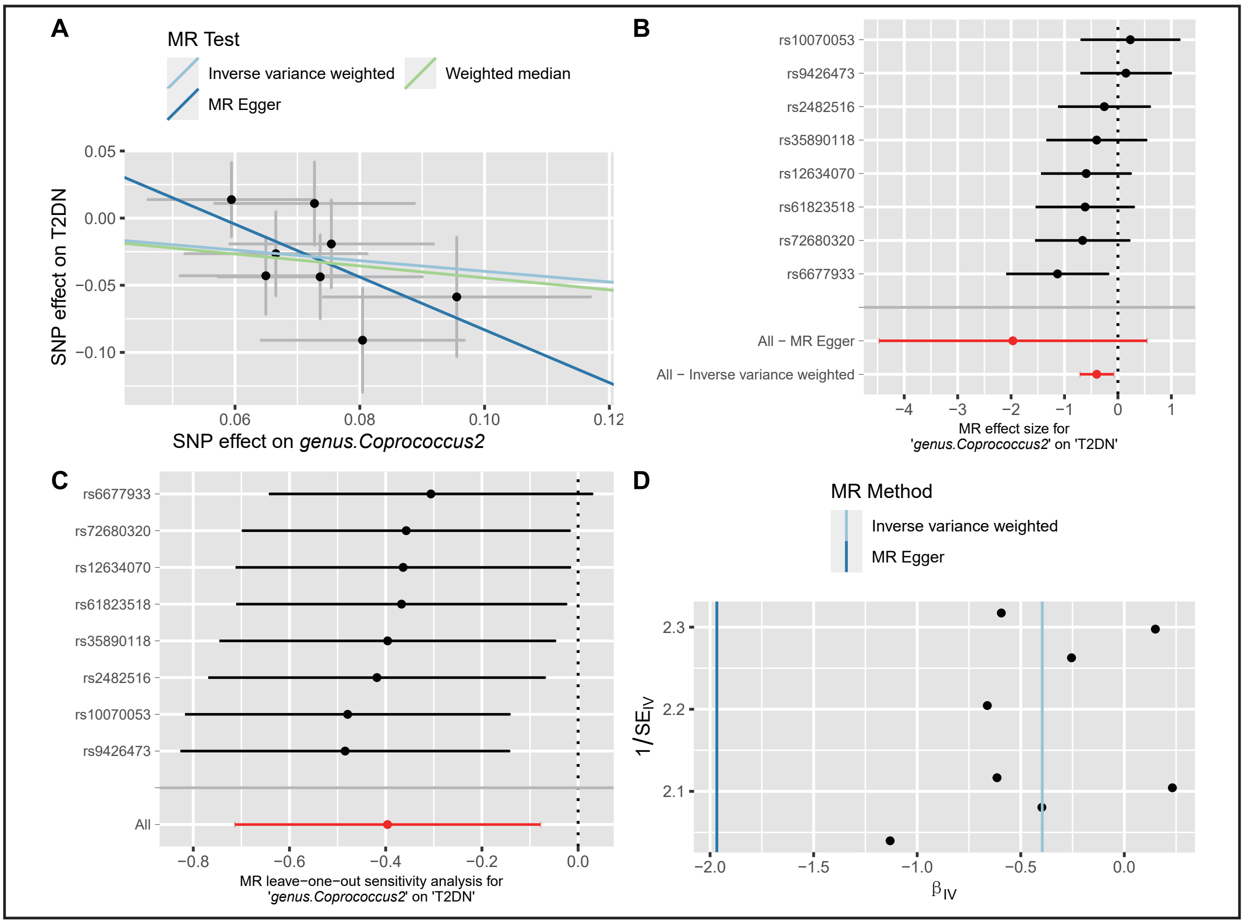

Figure S20 The visualisation results for describing the causal association between ***g_Coprococcus2*** and type 2 diabetic nephropathy. (A) Scatter plot; (B) Forest plot; (C) Leave-one-out sensitivity analysis plot; (D) Funnel plot.

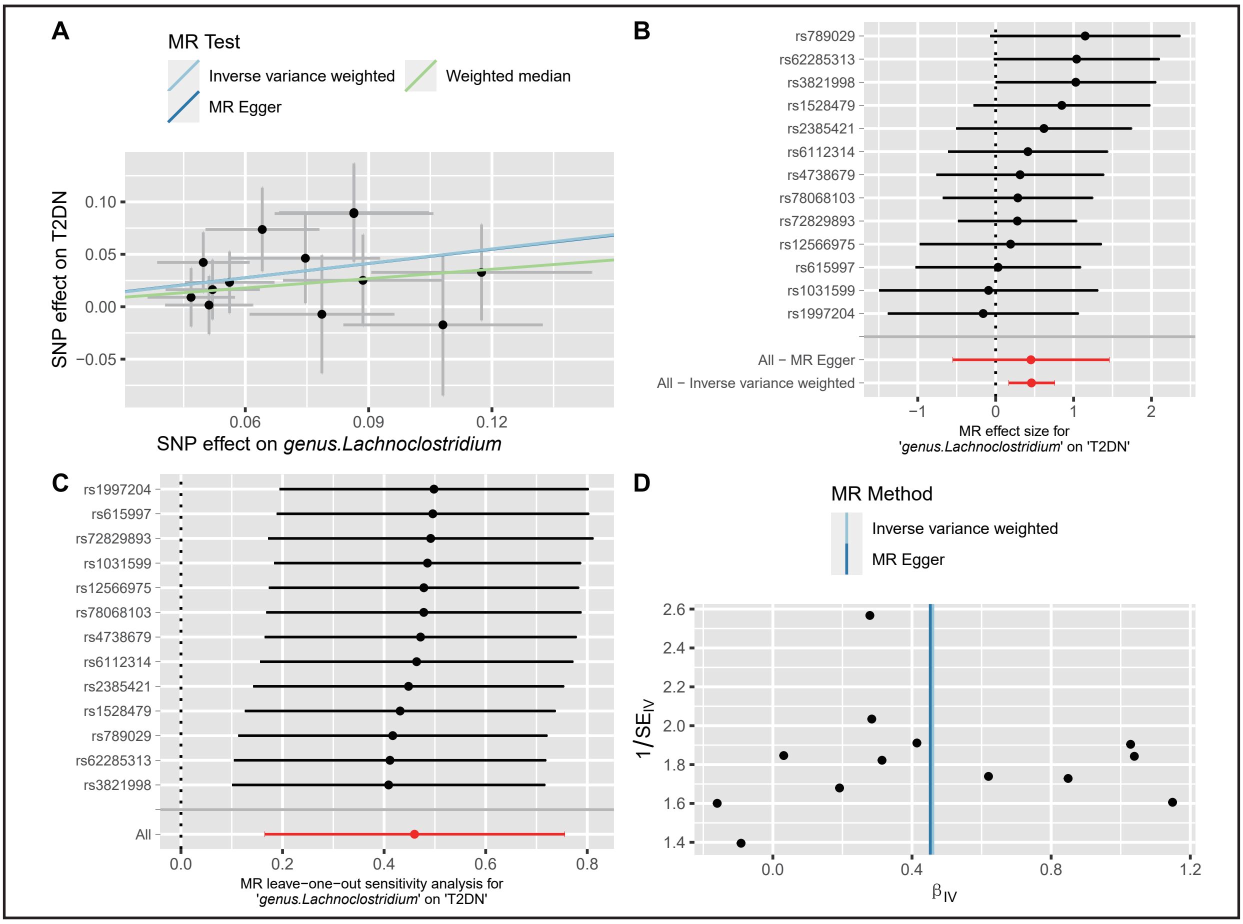

Figure S21 The visualisation results for describing the causal association between ***g_Lachnoclostridium*** and type 2 diabetic nephropathy. (A) Scatter plot; (B) Forest plot; (C) Leave-one-out sensitivity analysis plot; (D) Funnel plot.

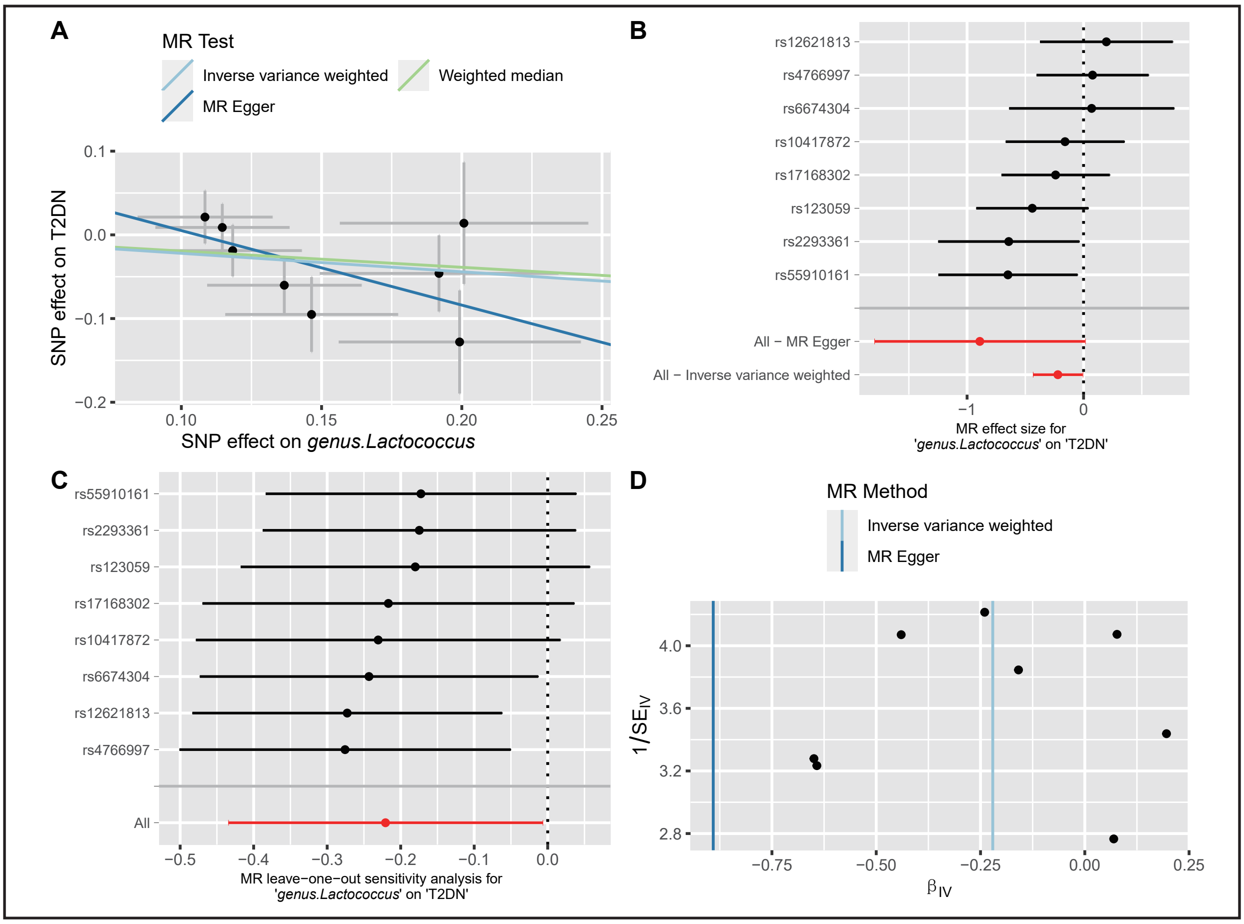

Figure S22 The visualisation results for describing the causal association between ***g_Lactococcus*** and type 2 diabetic nephropathy. (A) Scatter plot; (B) Forest plot; (C) Leave-one-out sensitivity analysis plot; (D) Funnel plot.

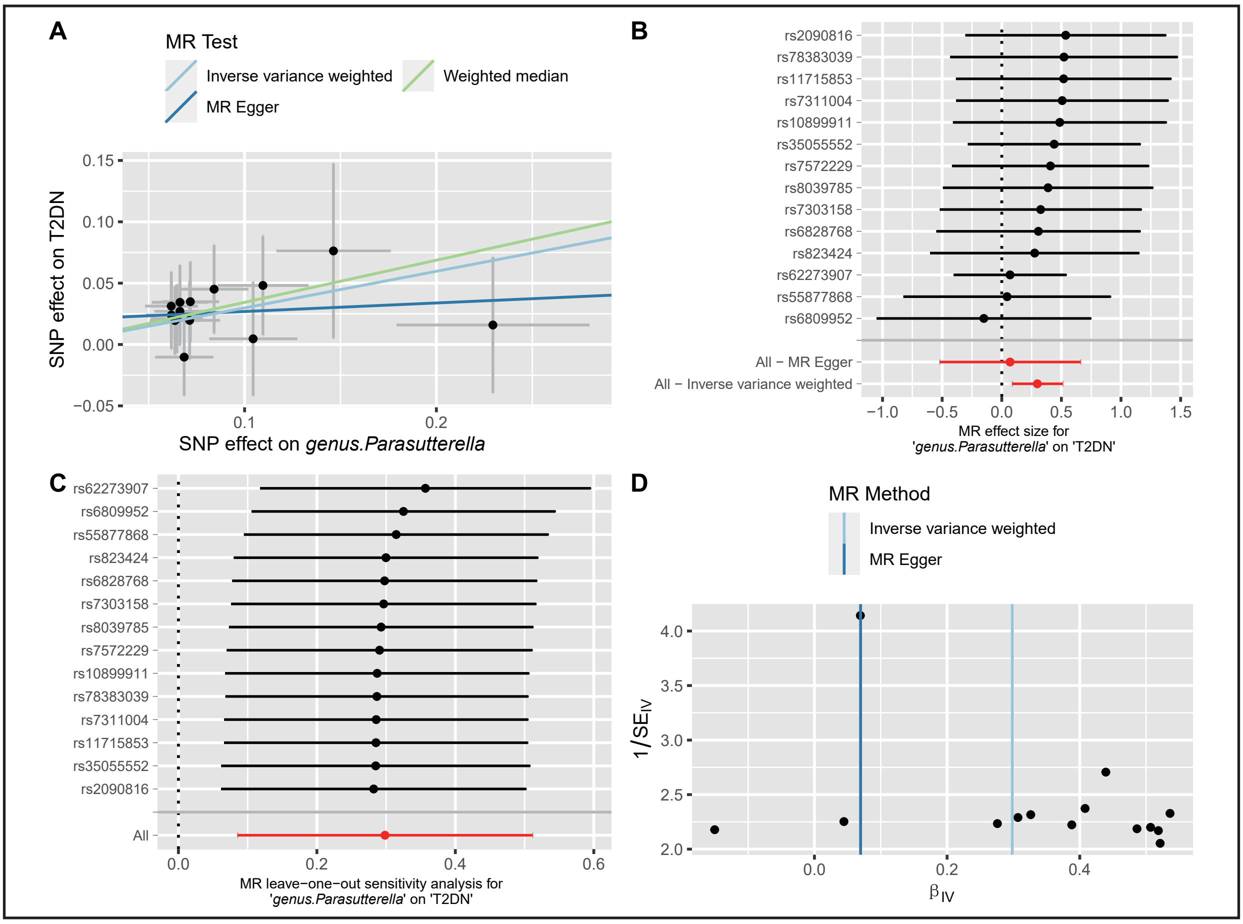

Figure S23 The visualisation results for describing the causal association between ***g_Parasutterella*** and type 2 diabetic nephropathy. (A) Scatter plot; (B) Forest plot; (C) Leave-one-out sensitivity analysis plot; (D) Funnel plot.

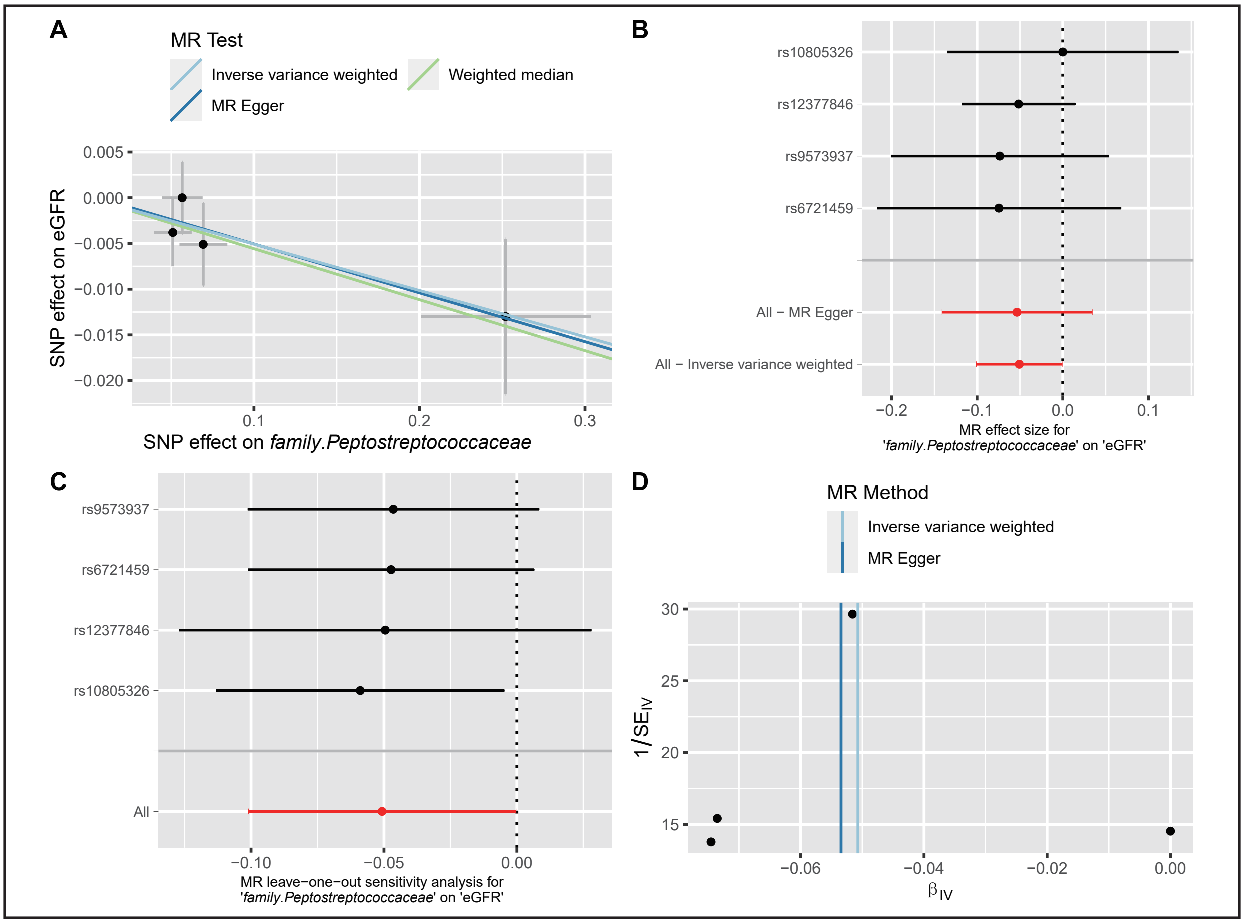

Figure S24 The visualisation results for describing the causal association between ***f_Peptostreptococcaceae*** and glomerular filtration rate (GFR). (A) Scatter plot; (B) Forest plot; (C) Leave-one-out sensitivity analysis plot; (D) Funnel plot.

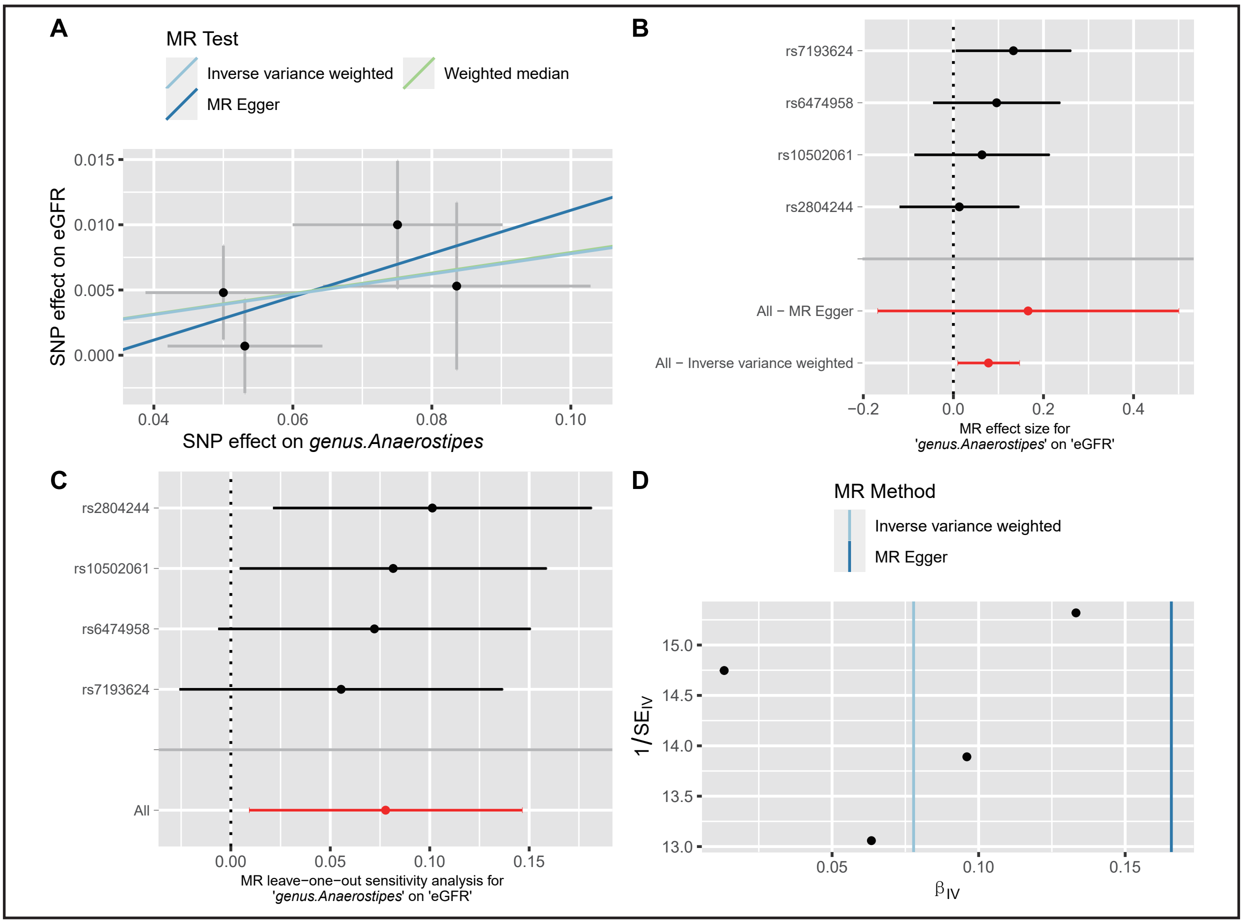

Figure S25 The visualisation results for describing the causal association between ***g_Anaerostipes*** and glomerular filtration rate (GFR). (A) Scatter plot; (B) Forest plot; (C) Leave-one-out sensitivity analysis plot; (D) Funnel plot.

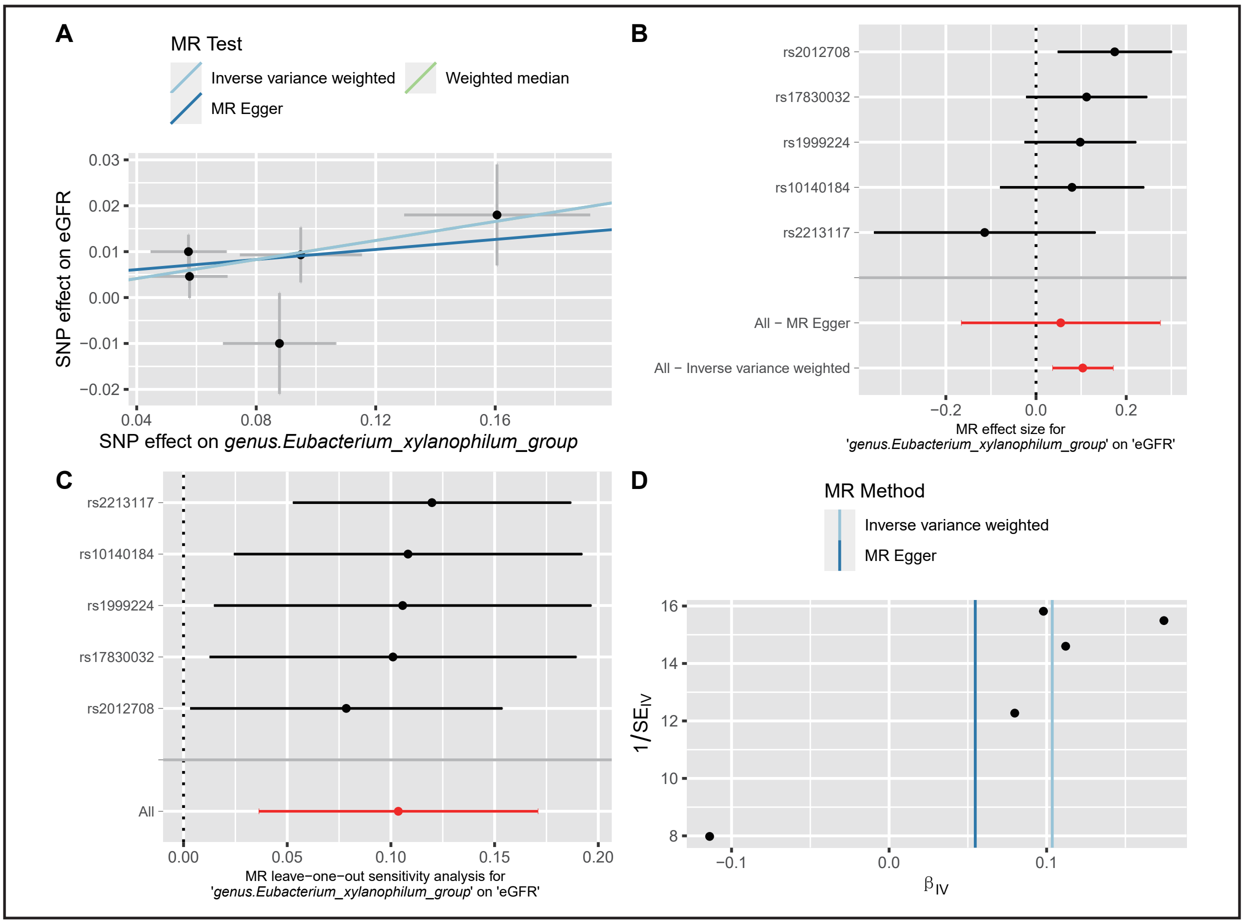

Figure S26 The visualisation results for describing the causal association between ***g_Eubacterium_xylanophilum_group*** and glomerular filtration rate (GFR). (A) Scatter plot; (B) Forest plot; (C) Leave-one-out sensitivity analysis plot; (D) Funnel plot.

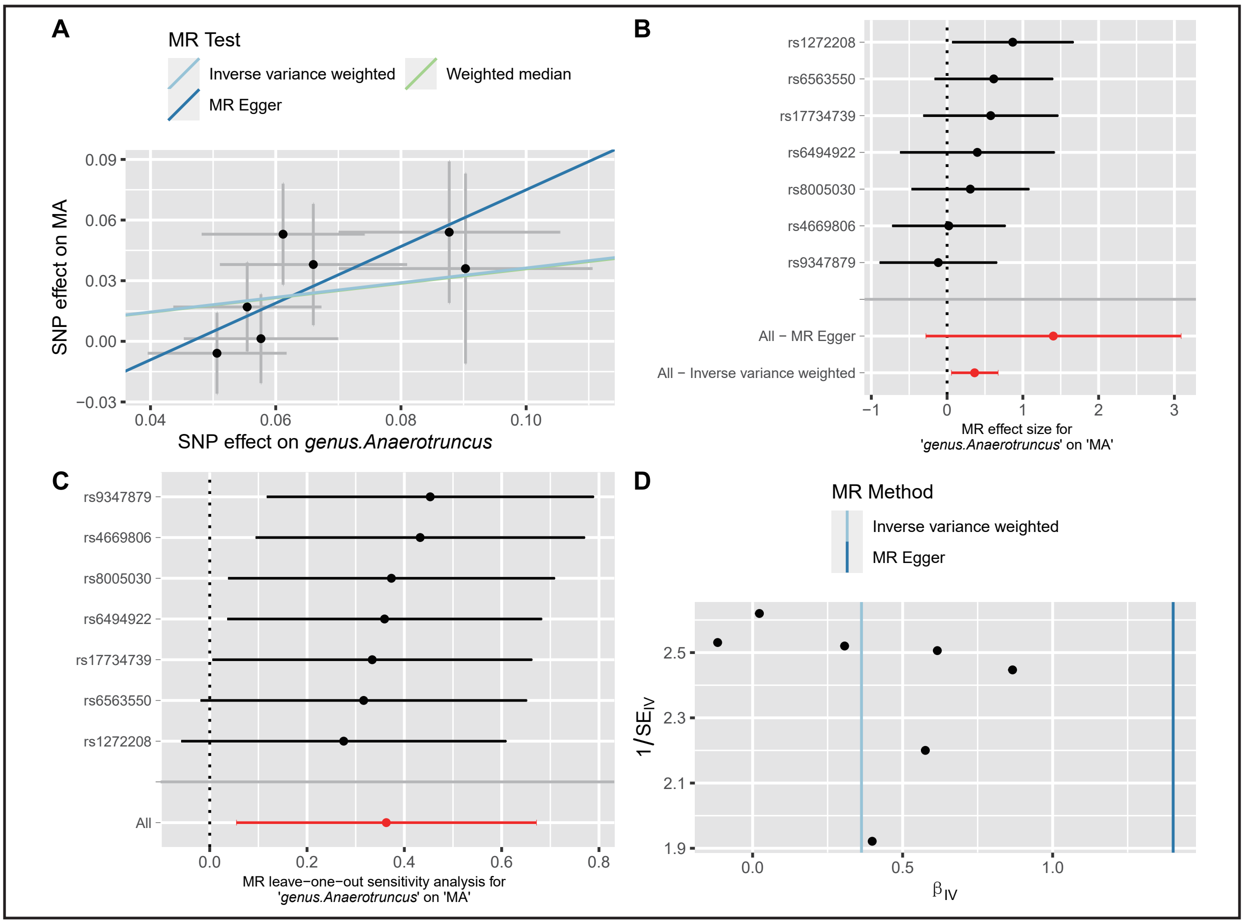

Figure S27 The visualisation results for describing the causal association between ***g_Anaerotruncus*** and microalbuminuria. (A) Scatter plot; (B) Forest plot; (C) Leave-one-out sensitivity analysis plot; (D) Funnel plot.

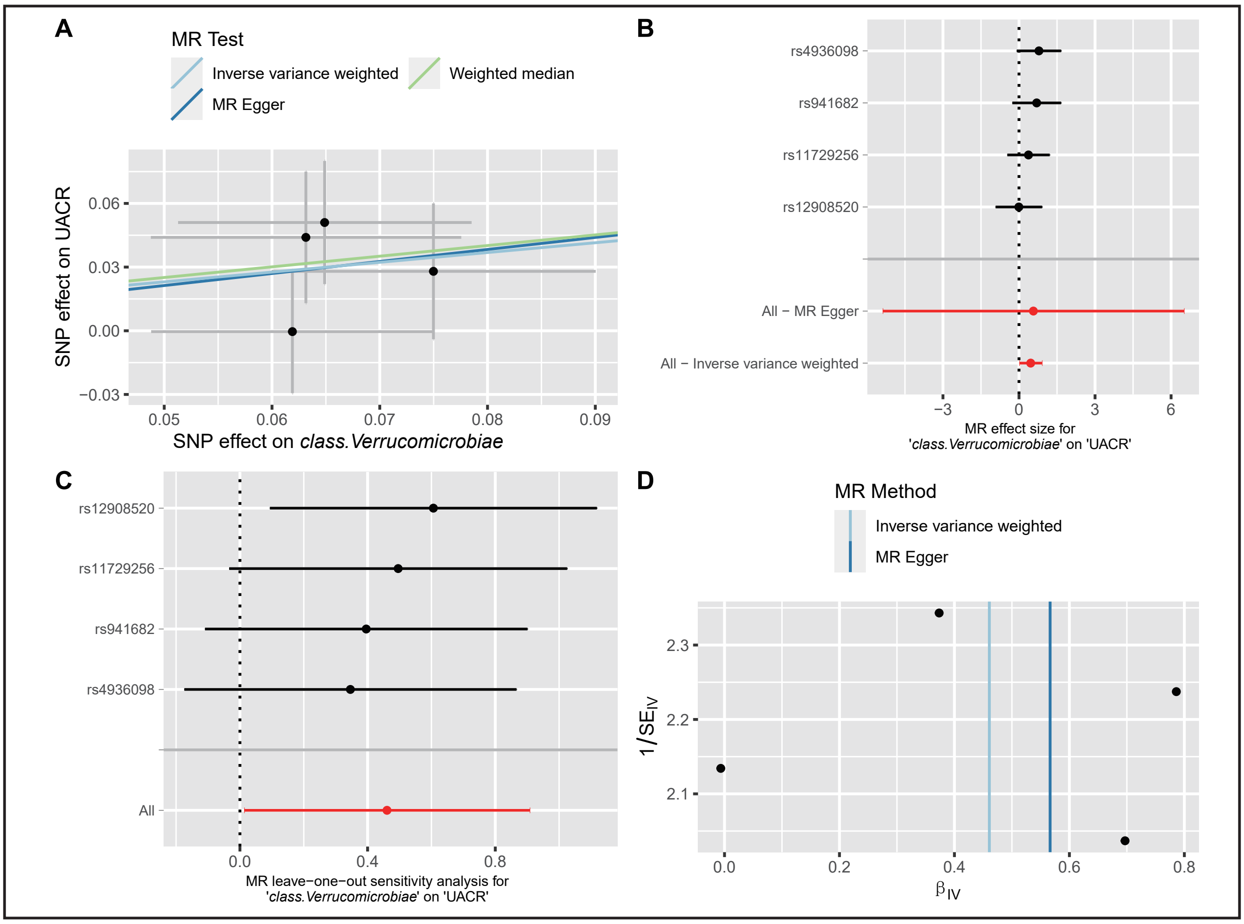

Figure S28 The visualisation results for describing the causal association between ***c_Verrucomicrobiae*** and urinary albumin to creatinine ratio (UACR). (A) Scatter plot; (B) Forest plot; (C) Leave-one-out sensitivity analysis plot; (D) Funnel plot.

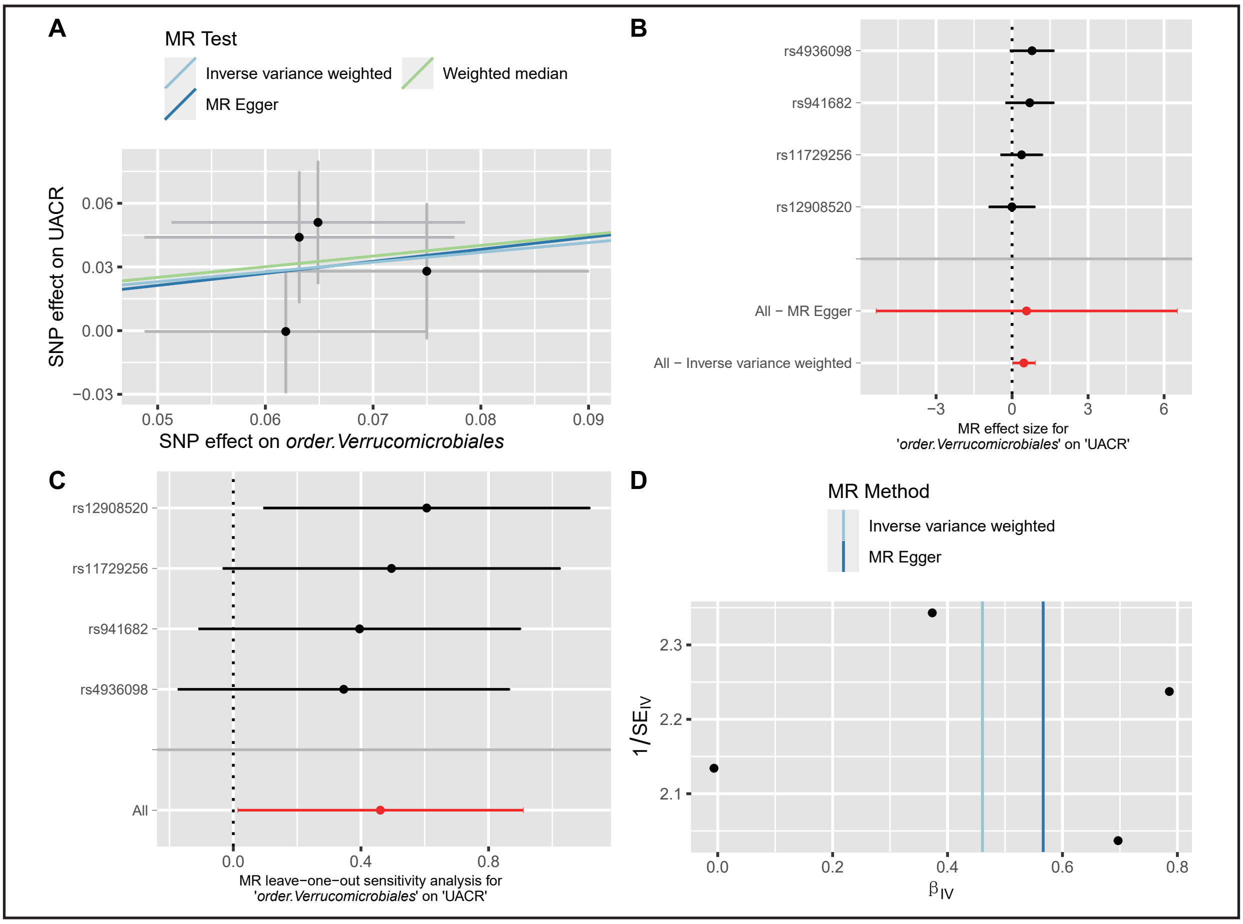

Figure S29 The visualisation results for describing the causal association between ***o_Verrucomicrobiales*** and urinary albumin to creatinine ratio (UACR). (A) Scatter plot; (B) Forest plot; (C) Leave-one-out sensitivity analysis plot; (D) Funnel plot.

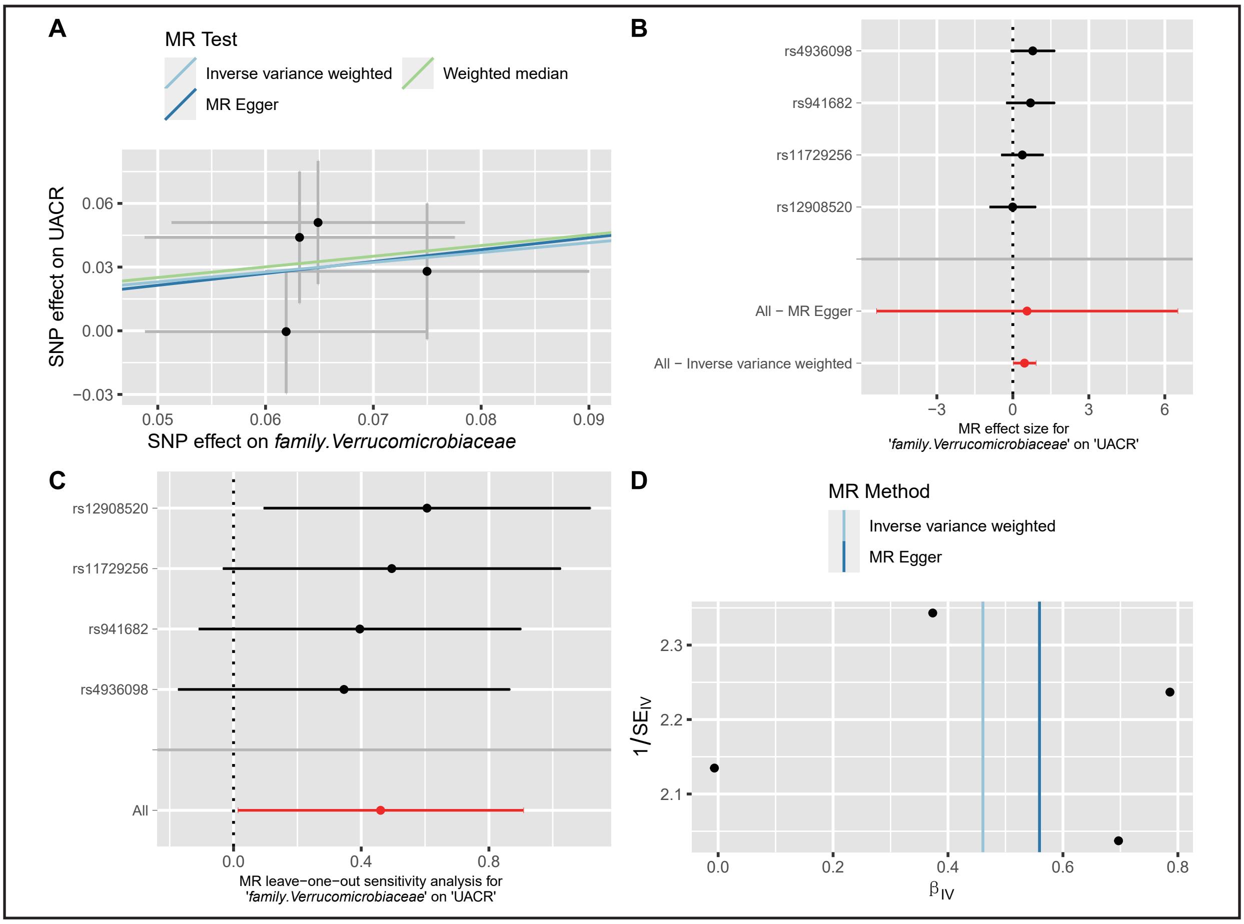

Figure S30 The visualisation results for describing the causal association between ***f_Verrucomicrobiaceae*** and urinary albumin to creatinine ratio (UACR). (A) Scatter plot; (B) Forest plot; (C) Leave-one-out sensitivity analysis plot; (D) Funnel plot.

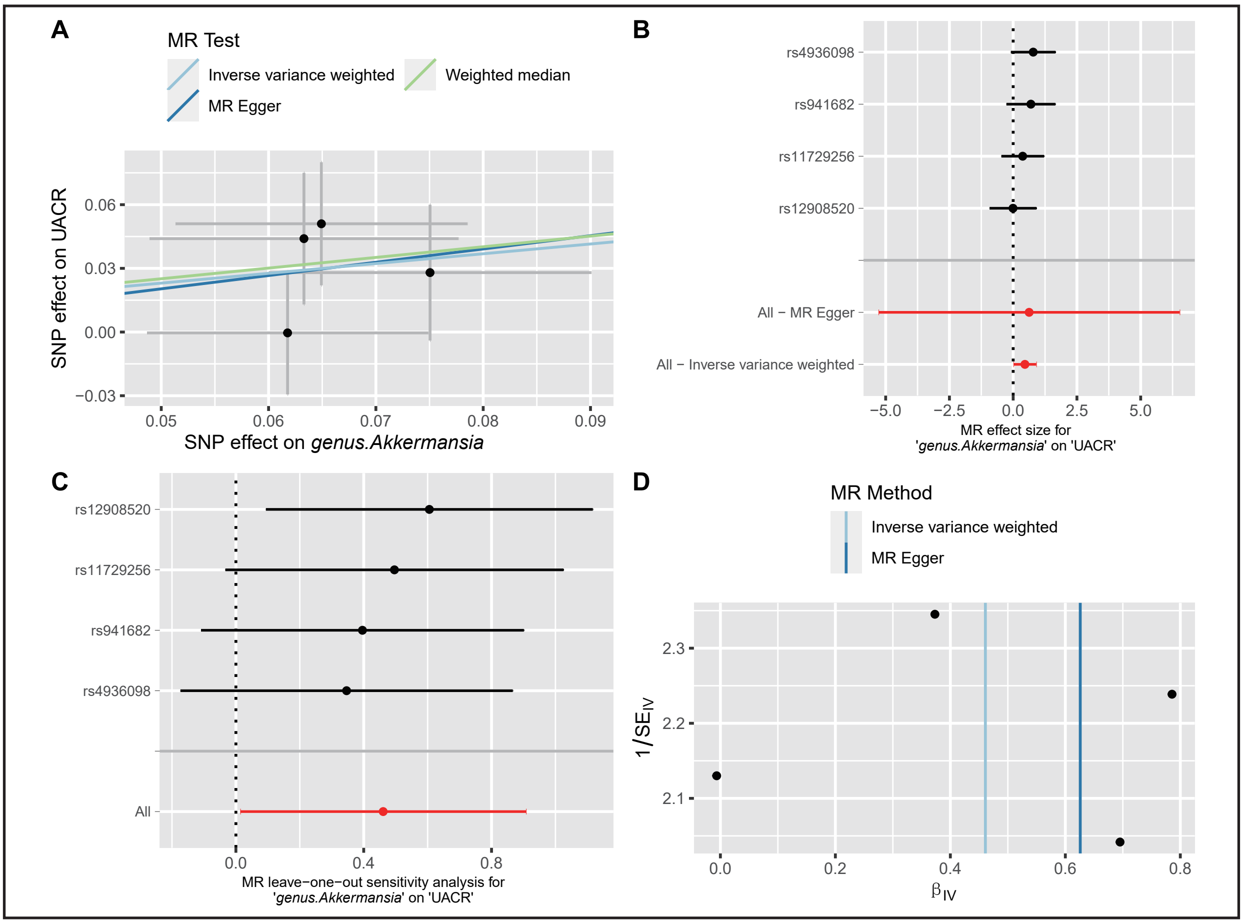

Figure S31 The visualisation results for describing the causal association between ***g_Akkermansia*** and urinary albumin to creatinine ratio (UACR). (A) Scatter plot; (B) Forest plot; (C) Leave-one-out sensitivity analysis plot; (D) Funnel plot.

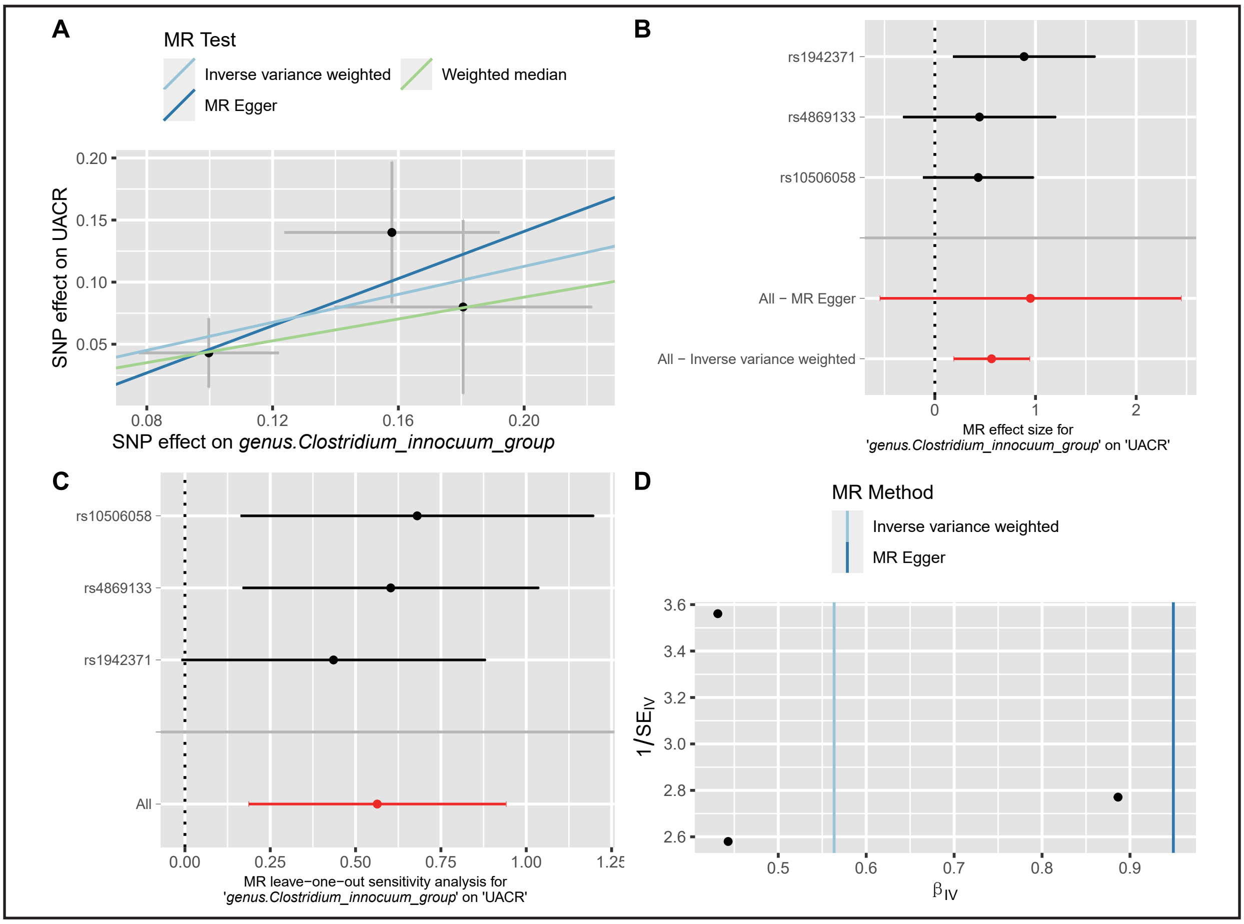

Figure S32 The visualisation results for describing the causal association between ***g_Clostridium_innocuum_group*** and urinary albumin to creatinine ratio (UACR). (A) Scatter plot; (B) Forest plot; (C) Leave-one-out sensitivity analysis plot; (D) Funnel plot.

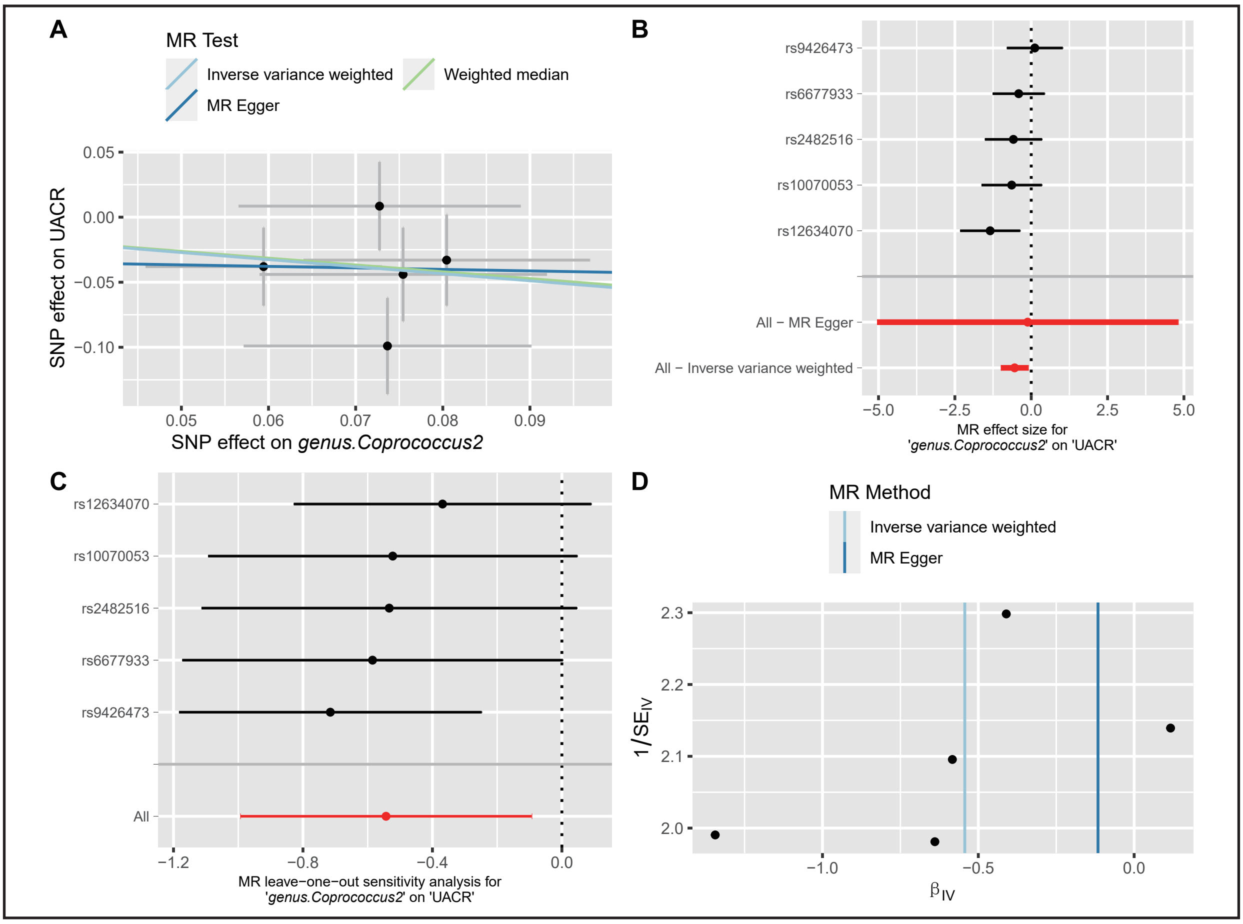

Figure S33 The visualisation results for describing the causal association between ***g_Coprococcus2*** and urinary albumin to creatinine ratio (UACR). (A) Scatter plot; (B) Forest plot; (C) Leave-one-out sensitivity analysis plot; (D) Funnel plot.

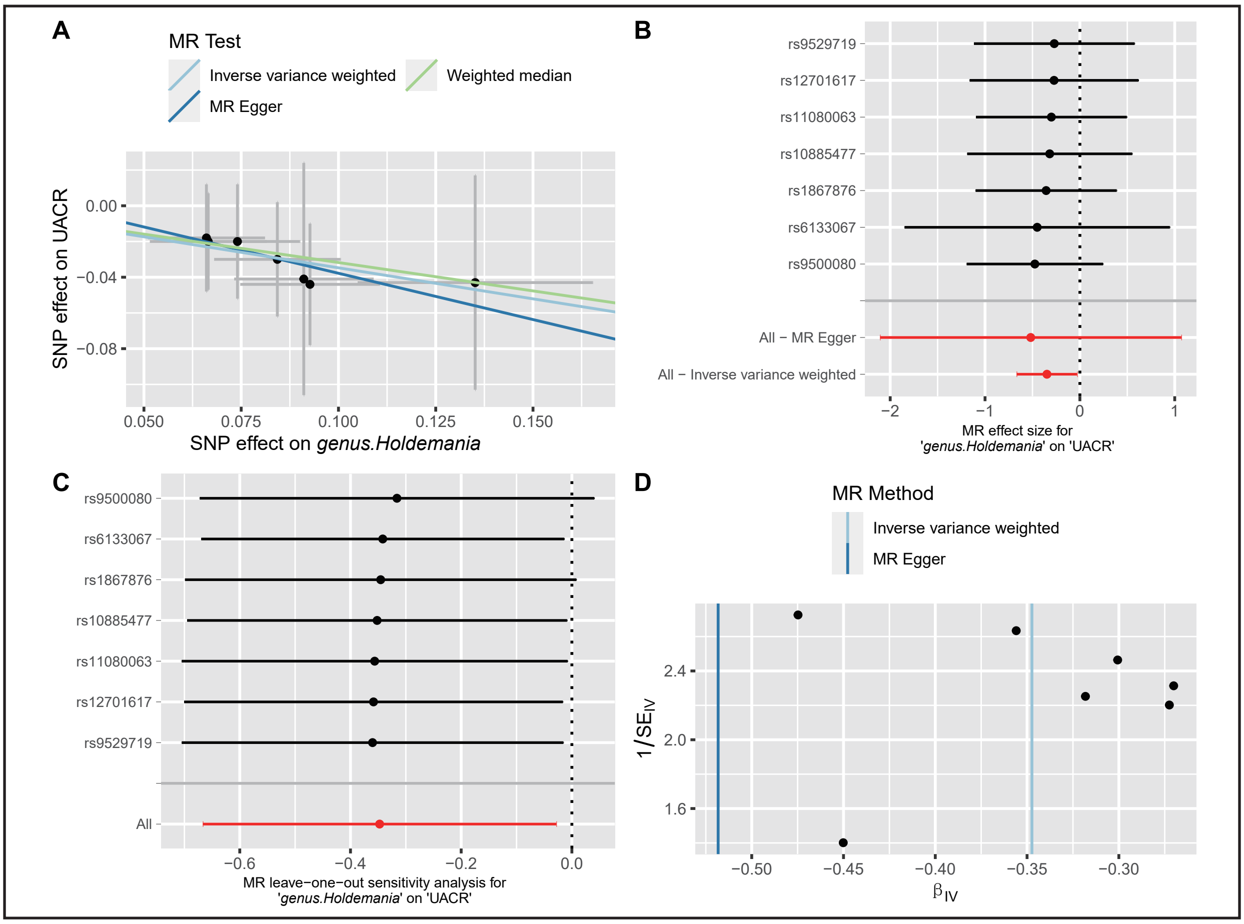

Figure S34 The visualisation results for describing the causal association between ***g_Holdemania*** and urinary albumin to creatinine ratio (UACR). (A) Scatter plot; (B) Forest plot; (C) Leave-one-out sensitivity analysis plot; (D) Funnel plot.

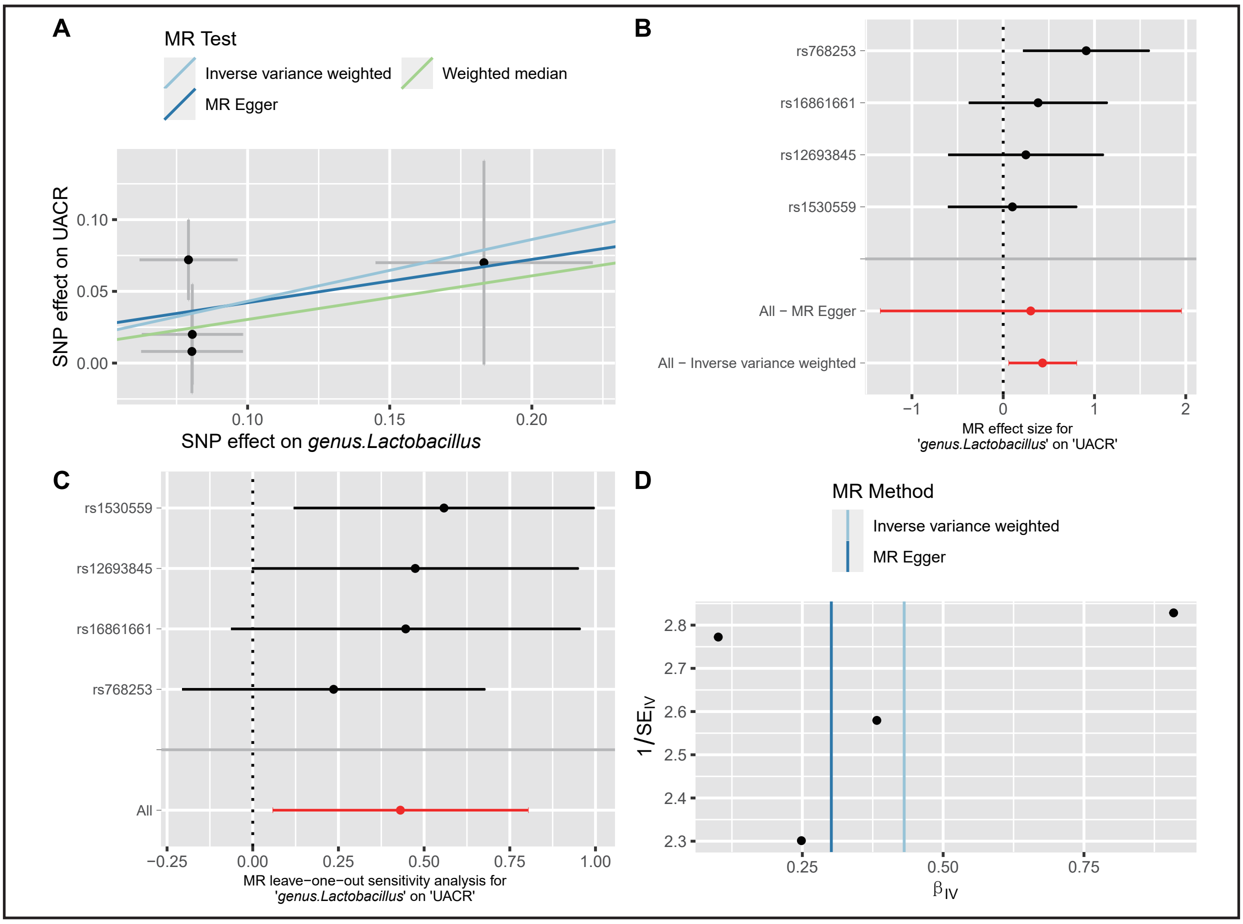

Figure S35 The visualisation results for describing the causal association between ***g_Lactobacillus*** and urinary albumin to creatinine ratio (UACR). (A) Scatter plot; (B) Forest plot; (C) Leave-one-out sensitivity analysis plot; (D) Funnel plot.
