# Supplementary material for: Assessing the causal relationship between gut microbiota and diabetic nephropathy: insights from two-sample Mendelian randomization
Source: Front Endocrinol (Lausanne). 2024 Mar 18;15:1329954. doi: 10.3389/fendo.2024.1329954 (PMC10982433; doi:10.3389/fendo.2024.1329954)
Supplement: Supplementary file 2 [file DataSheet_2.pdf]

Assessing the Causal Relationship between Gut Microbiota and Diabetic Nephropathy: Insights from Two-Sample Mendelian Randomization

Supplemental file 2 The distinct analysis code for present study.

```
rm(list=ls())
#===Import local external GWAS data of DN===
# GWAS data of DN should be download from FinngenR9 and CKDGen databases
DN_gwas<-data.table::fread("..\\..\\DN
GWAS\\finngen_R9_DM_NEPHROPATHY_EXMORE.gz")
DN_gwas<-data.frame(DN_gwas)
head(DN_gwas)
# Modify the column names
colnames(DN_gwas)[c(4,3,5,7,9,10,11)]<-c("effect_allele.outcome","other_allele.outcome",
"SNP","pval.outcome","beta.outcome","se.outcome","eaf.outcome")
# Add the column names
DN_gwas$id.outcome<-"DN"
DN_gwas$outcome<-"DN"
DN_gwas$samplesize.outcome<-312650
# Retain SNPs unrelated to outcome, pval.outcome>5e-08
DN_gwas<-subset(DN_gwas,pval.outcome>5e-08)

#===Import local external GWAS data of GM===
# GWAS data of GM should be download from MiBioGen databases
exp <- read.csv("..\\..\\data of GM from MiBioGen.csv")
colnames(exp) <- c("id.exposure", "chr", "pos", "SNP",
                   "other_allele.exposure", "effect_allele.exposure",
                   "beta.exposure", "se.exposure", "meta", "pval.exposure",
                   "N", "nn")
exp$exposure <- exp$id.exposure
data_class <- unique(exp$id.exposure)
dir.create("GM_data_for_analysis")
for (i in data_class) {
  names <- subset(exp, id.exposure == i)
  cfilename = paste0("GM_data_for_analysis\\",
```

```
                                   i, ".csv")
    write.csv(names, cfilename)
}
cat("Finish")


#===MR analysis===
savefile <- "Result of analysis"
outcome <- "DN"
library(TwoSampleMR)
dir.create(savefile)
filename <- data.frame(dir("GM_data_for_analysis\\"))
A_temp <- c()
B_temp <- c()
C_temp <- c()
D_temp <- c()
for (i in filename[, 1]) {
    ipath <- paste0("GM_data_for_analysis\\", "\\", i)
    exp_temp <- read.csv(ipath, header = T)
    test2 <- (try(exp_temp <- clump_data(exp_temp, clump_kb = 10000,
                                          clump_r2 = 0.001)))
    if (class(test2) == "try-error") {
        total <- merge(DN_gwas, exp_temp, by = "SNP")
        total$eaf.exposure <- NA    #total$eaf.exposure <- total$eaf.outcome
        exp3 <- total[, c("SNP", "effect_allele.exposure",
                              "other_allele.exposure", "beta.exposure", "se.exposure",
                              "pval.exposure", "id.exposure", "exposure",
                              "eaf.exposure")]
        out3 <- total[, c("SNP", "effect_allele.outcome",
                              "other_allele.outcome", "eaf.outcome", "beta.outcome",
                              "se.outcome", "pval.outcome", "id.outcome",
                              "outcome", "eaf.outcome")]
        dat <- harmonise_data(exposure_dat = exp3, outcome_dat = out3,
                                  action = 2)
        data_h<-dat%>%subset(dat$mr_keep==TRUE)
        data_h$Fvalue                                                    <-
```

```
(data_h$beta.exposure/data_h$se.exposure)*(data_h$beta.exposure/data_h$se.exposure)
    data_h_TableS1 <- data_h[, c("exposure","SNP","effect_allele.exposure",
"other_allele.exposure",
                    "beta.exposure",
"se.exposure","Fvalue","pval.exposure",
                    "beta.outcome","se.outcome", "pval.outcome")]
    data_h_TableS1$cluster <- 0
    res <- mr(data_h)
    res$cluster <- 0
    mr_OR<-generate_odds_ratios(res)
    mr_OR$or<-round(mr_OR$or,3)
    mr_OR$or_lci95<-round(mr_OR$or_lci95,3)
    mr_OR$or_uci95 <- round(mr_OR$or_uci95,3)
    mr_OR$OR_CI <- paste0(mr_OR$or,"(",mr_OR$or_lci95,"-",mr_OR$or_uci95,")")
  }
  else {
    total <- merge(DN_gwas, exp_temp, by = "SNP")
    total$eaf.exposure <- NA    #total$eaf.exposure <- total$eaf.outcome
    exp3 <- total[, c("SNP", "effect_allele.exposure",
                  "other_allele.exposure", "beta.exposure", "se.exposure",
                  "pval.exposure", "id.exposure", "exposure",
                  "eaf.exposure")]
    out3 <- total[, c("SNP", "effect_allele.outcome",
                  "other_allele.outcome", "eaf.outcome", "beta.outcome",
                  "se.outcome", "pval.outcome", "id.outcome",
                  "outcome", "eaf.outcome")]
    dat <- harmonise_data(exposure_dat = exp3, outcome_dat = out3,
                    action = 2)
    data_h<-dat%>%subset(dat$mr_keep==TRUE)
    data_h$Fvalue <-
(data_h$beta.exposure/data_h$se.exposure)*(data_h$beta.exposure/data_h$se.exposure)
    data_h_TableS1 <- data_h[, c("exposure","SNP","effect_allele.exposure",
"other_allele.exposure",
                    "beta.exposure",
"se.exposure","Fvalue","pval.exposure",
                    "beta.outcome","se.outcome", "pval.outcome")]
```

```
        data_h_TableS1$cluster <- 1
        res <- mr(data_h)
        res$cluster <- 1
        mr_OR<-generate_odds_ratios(res)
        mr_OR$or<-round(mr_OR$or,3)
        mr_OR$or_lci95<-round(mr_OR$or_lci95,3)
        mr_OR$or_uci95 <- round(mr_OR$or_uci95,3)
        mr_OR$OR_CI <- paste0(mr_OR$or,"(",mr_OR$or_lci95,"-",mr_OR$or_uci95,")")
    }
    if (dim(res)[[1]] != 0) {
        het <- mr_heterogeneity(dat)
        ple <- mr_pleiotropy_test(dat)
        A_temp <- rbind(mr_OR, A_temp)
        B_temp <- rbind(het, B_temp)
        C_temp <- rbind(ple, C_temp)
        D_temp <- rbind(data_h_TableS1, D_temp)
        print(paste0( i, "is running"))
        Aname <- paste0(savefile, "\\", "01.MR analysis between GM and ",
                                outcome, ".csv")
        Bname <- paste0(savefile, "\\", "02.heterogeneity analysis between GM and ",
                                outcome, ".csv")
        Cname <- paste0(savefile, "\\", "03.pleiotropy analysis between GM and ",
                                outcome, ".csv")
        Dname <- paste0(savefile, "\\", "04.SNPs between GM and ",
                                outcome, ".csv")
        write.csv(A_temp, Aname, row.names = F)
        write.csv(B_temp, Bname, row.names = F)
        write.csv(C_temp, Cname, row.names = F)
        write.csv(D_temp, Dname, row.names = F)
    }
    else {
        cat("the GM_data of",
            i, "should be removed")
    }
}
cat("MR analysis over")
```
